# Regulating glycerol electrooxidation pathways *via* catalytic environments

**DOI:** 10.1039/d6cs00908e

**Published:** 2026-11-05

**Authors:** Yabo Qi, Hanzhi Ye, Shouxin Liu, Hui Luo, Hongwu Chen, Maria-Magdalena Titirici

**Affiliations:** a Department of Chemical Engineering, Imperial College London South Kensington Campus London SW7 2AZ UK m.titirici@imperial.ac.uk; b State Key Laboratory of Woody Oil Resources Utilization, Northeast Forestry University Harbin 150040 China liushouxin@126.com; c Research Center of Renewable Energy, SINOPEC Research Institute of Petroleum Processing Beijing 100083 China chenhongwu.ripp@sinopec.com; d School of Engineering, University of Surrey Guildford GU2 7XH UK hui_luo@surrey.ac.uk

## Abstract

Electrocatalytic transformation of abundant organic molecules to value-added chemicals has emerged as a promising pathway towards building a sustainable chemical industry using renewable electricity. As a byproduct of biodiesel production, glycerol can be oxidized electrochemically to synthesize a variety of value-added C1–C3 chemicals including glyceric acid, lactic acid, dihydroxyacetone, glycolic acid and formic acid *etc*. However, practical glycerol electrooxidation is challenged by limited product selectivity, insufficient activity and poor catalyst stability, which are deeply rooted in the reaction pathway including adsorption, electrochemical dehydrogenation, chemical transformation of intermediates, C–C bond cleavage and product desorption. This review organizes current mechanistic understanding around these coupled steps and examines how catalytic environments, comprising catalyst composition, interfacial properties and reaction conditions, regulate reaction pathways. We elucidate the critical factors that determine the reaction activity and selectivity towards retained C3 or C1/C2 products. Additionally, we distinguish site blocking caused by strongly adsorbed species or slow desorption from deactivation associated with potential-induced surface oxidation and structural evolution. The active-site engineering, regulation of interfacial structure and reaction conditions are connected to the optimisation of electrochemical performance *via* reactant activation, controlled intermediate transformation, organic residue removal and surface regeneration. This step-resolved perspective provides mechanistic guidance for designing efficient, selective and durable glycerol electrooxidation systems.

## Abstract 2

A step-resolved framework explains how catalytic environments regulate adsorption, dehydrogenation, chemical conversion, C–C cleavage and desorption to shape glycerol electrooxidation selectivity, activity and stability.

## Graphical abstract

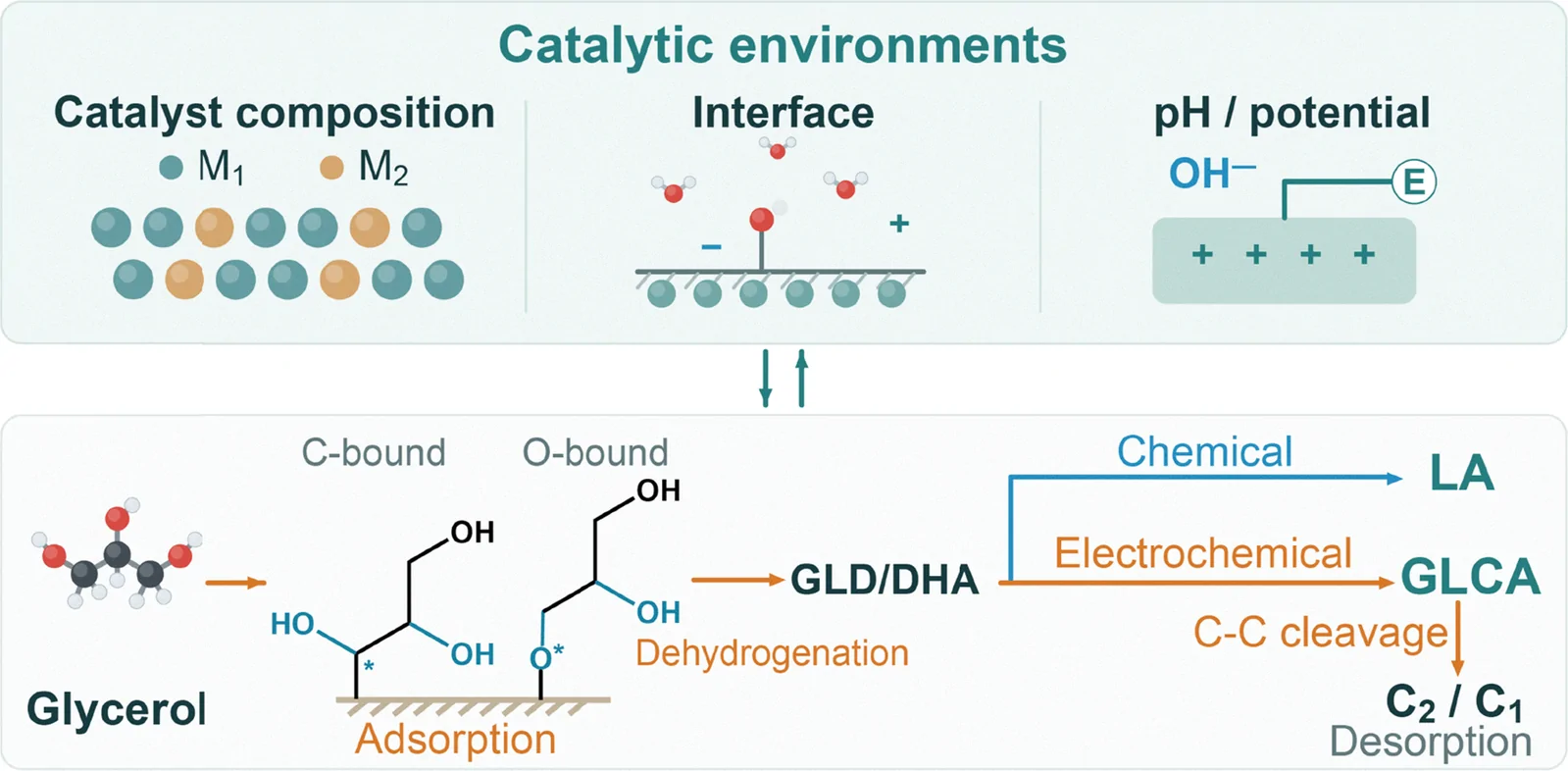

## 1. Introduction

The International Energy Agency (IEA) reported that renewables and nuclear power accounted for 80% of global electricity growth in 2024.^1^ This ongoing transition to renewable electricity, however, also introduces the challenge of curtailment, in which excess renewable generation is deliberately discarded to maintain grid stability. Although operationally necessary, curtailment reduces the utilization of renewable electricity and undermines the economic competitiveness of renewable technologies.^2^ In this context, electrocatalysis offers a promising Power-to-X (P2X) strategy by converting surplus electricity into chemicals stored in chemical bonds.^3,4^ Among such electrochemical reactions, various cathodic reactions including hydrogen evolution reaction (HER) and electrochemical CO_2_ reduction reaction (CO_2_RR) are promising half-reactions that produce “green hydrogen” and syngas/ethylene for establishing an electrified and more sustainable chemical industry.^5–8^ However, the coupled anodic reaction – generally the oxygen evolution reaction (OER), is sluggish, with a theoretical thermodynamic potential around 1.23 V against reversible hydrogen electrode (RHE), which is energy consuming and less profitable as oxygen could be easily separated from the air.^9^ Therefore, alternative anodic reactions (AARs) have been proposed over the past decades as a strategy to reduce the energy consumption and produce value-added chemicals.^10–12^

A variety of reaction substrates were identified for AARs, ranging from bio-derived chemicals to fossil-fuel based derivatives such as polyethylene terephthalate (PET) directly or PET derived ethylene glycol.^13^ Among them, glycerol is an abundant feedstock generated as a byproduct of biodiesel production, with a market size of about 4 million tons per year.^14^ Depending on the catalyst and reaction conditions, glycerol electrooxidation reaction (GOR) can proceed through oxidation of the hydroxyl groups and/or C–C bond cleavage, yielding a series of C1–C3 products including lactic acid (LA, C3), glyceric acid (GLCA, C3), tartronic acid (TA, C3), mesoxalic acid (MA, C3), dihydroxyacetone (DHA, C3), glycolic acid (GCA, C2), oxalic acid (OA, C2) and formic acid (FA, C1).^15–18^ Most of the above major products have chemical industrial importance. LA is the building block of biodegradable plastics (polylactic acid), which is critical for the sustainable transition to biodegradable packaging.^19–21^ GLCA and TA serve as biodegradable surfactants and emulsifiers, or chelating agents in the market of daily chemicals.^22–24^ DHA and GCA have wide applications in cosmetics, as DHA is a self-tanning agent targeting the amino acids of the cells of the stratum corneum and GCA is commonly used as a chemical exfoliation agent which improves the skin health and appearance.^25–27^ Though FA is a C1 chemical produced by the down-cycling of glycerol, the application in preservative, antibacterial agent, leather tanning and hydrogen carrier renders it as a useful chemical.^28,29^ However, as summarized in Fig. 1, these products differ substantially in their techno-economic positions with respect to product price, market revenue, and projected annual profit. Within this simplified screening, glyceraldehyde (GLD) appears particularly favourable because of its high reported price and market revenue. However, this should not be taken as evidence that GLD is necessarily the most practical target product, since its instability and the associated challenges in handling and purification may limit its practical attractiveness despite its apparent economic promise. Because the underlying analysis assumes single-product formation, 100% selectivity and faradaic efficiency, and excludes separation, electricity, and overhead costs, Fig. 1 should be interpreted as a qualitative screening map rather than a direct ranking of practical target products. More generally, products that retain the C3 backbone tend to occupy more favourable positions, whereas further oxidation and downstream C–C bond cleavage to C2 or C1 products do not necessarily deliver greater economic benefit. These differences emphasize that controlling GOR selectivity is important not only for product separation and catalytic efficiency, but also for maximizing the economic value of glycerol valorisation.

**Fig. 1:**
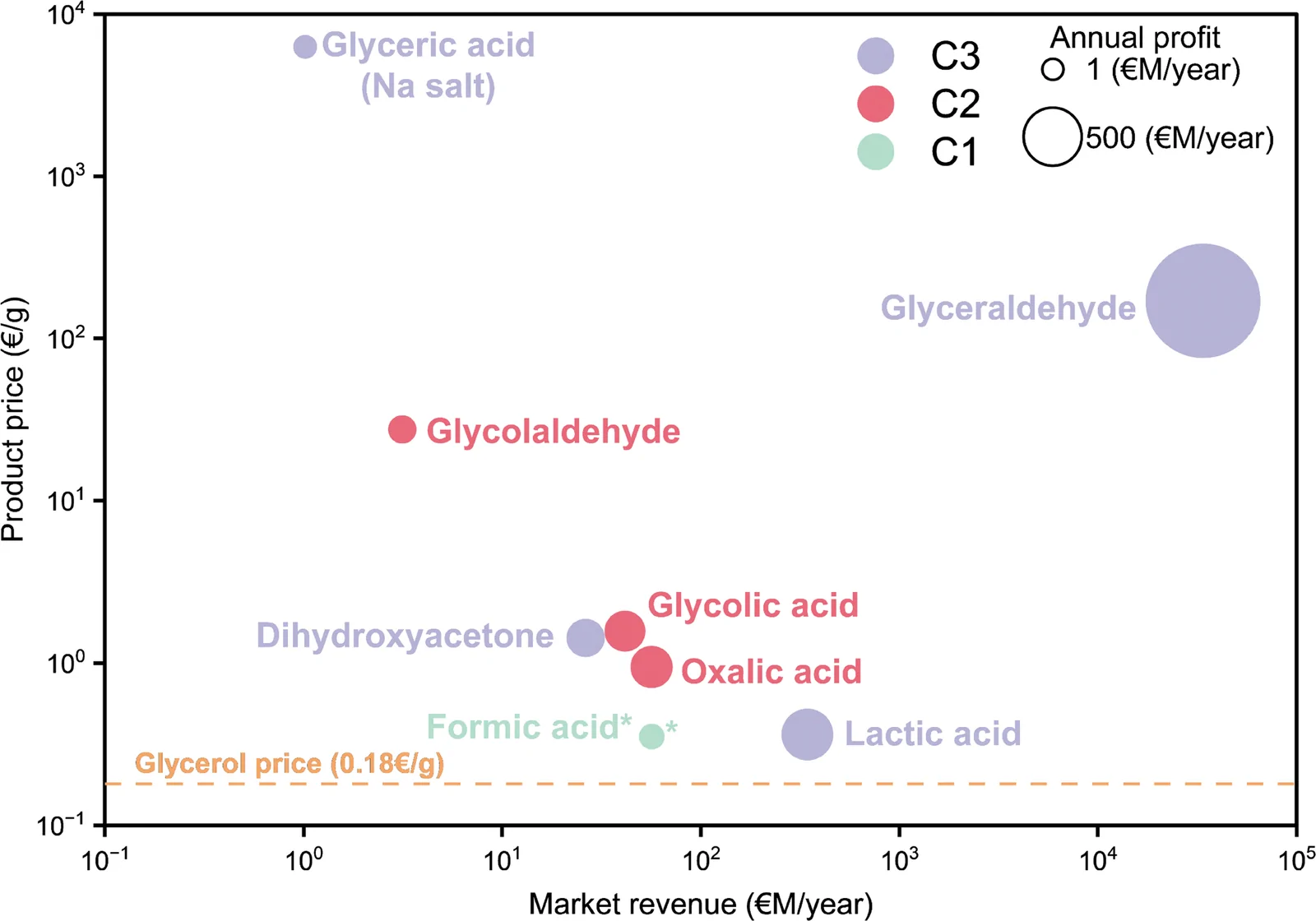
Economic positioning map of glycerol electrooxidation products. Products are coloured according to carbon number (C3, C2, and C1), and the horizontal dashed line marks the glycerol feedstock price (€0.18 g^−1^). Bubble size scales with the projected annual profit, such that larger bubbles indicate greater expected economic return. An asterisk is used to denote that formic acid shows a negative projected annual profit in the simplified analysis. Data were all compiled from Brennan-Pollak *et al.*^30^ and are intended for qualitative comparison only, as the original model assumes single-product formation, 100% selectivity and faradaic efficiency, and excludes separation, electricity, and overhead costs. TA is not shown because market-revenue data were unavailable in the source table, precluding a consistent placement on the current price-market map.

In the past decades, various noble-metal and earth-abundant metal-based catalysts were developed for the electrochemical valorisation of glycerol to the above chemicals.^17,31–34^ However, the practical use of such electrocatalytic systems relies on improved performance, which is manifested in the selectivity, activity and stability of the reaction. High selectivity is important because glycerol oxidation often produces a wide range of chemicals, making the separation of the target product more difficult and expensive.^35^ The activity of a catalytic system is governed by fundamental surface processes, such as the adsorption of glycerol and intermediates, the formation of reactive OH species (OH*), and the kinetics of competing oxidation pathways. Durability depends on the ability of the catalyst surface to resist deactivation during operation, including poisoning by strongly adsorbed intermediates, oxide formation, and structural changes of active sites.

Previous studies have explored various strategies to improve the selectivity, activity and durability of the GOR, such as modulating the electronic and geometric structures of the active sites^17,36–38^ (*e.g.*, *via* alloying, surface decoration, or defect engineering) and constructing synergistic catalyst–support interfaces^39–41^*etc*. These strategies have significantly enhanced GOR performance, bringing it closer to practical application. While conventional alkaline water electrolyzers typically operate at current densities of 200–400 mA cm^−2^,^6^ recent benchmarks for industrial relevance in GOR have shifted to exceeding 300 mA cm^−2^, with state-of-the-art catalysts now demonstrating stable operation at 500–800 mA cm^−2^ and peak densities surpassing 1000 mA cm^−2^.^42–46^ Nonetheless, the synergistic enhancement of a catalyst performance in all the performance indicators (activity, selectivity and stability) is inherently challenging. For example, strategies that enhance activity often focus on lowering the activation energy for initial dehydrogenation or decreasing the potential required to generate surface oxidants (*e.g.*, OH*). However, depending on the intrinsic nature of the catalyst, enhancing its oxidative power or strengthening its binding affinity for intermediates often results in over-oxidation, leading to the undesired cleavage of C–C bonds and lowering the selectivity towards specific C3 chemicals.^47^ To address this “activity–selectivity–stability” trilemma, a fundamental mechanistic understanding is required. Building on previous reviews of GOR,^12,17,31,48–52^ this review adopts a step-resolved framework that relates the catalytic environment to individual reaction steps and pathway branching. Here, the “catalytic environment” refers to the catalyst surface (composition, ensemble geometry and potential-dependent working oxidation state), the interface (adsorbate coverage and catalyst–support interactions), and the operating conditions (applied potential and the electrolyte environment, including composition, coordinating species and bulk or local pH). Because most mechanistic and catalyst-design studies have been conducted in alkaline electrolytes, alkaline systems provide the main evidence base of this review. Evidence from neutral and acidic media is also included to examine how pH-dependent changes in glycerol deprotonation, hydroxyl availability, catalyst surface state and intermediate stability affect C3 retention and C–C cleavage. We first introduce the overall reaction network of GOR and outline the main pathways leading to different products. We then assess the theoretical and experimental evidence supporting these pathway assignments. Sections 4–8 use a step-resolved framework to examine how catalyst structure, interfacial properties, applied potential and electrolyte conditions regulate adsorption (Section 4), dehydrogenation (Section 5), chemical transformations (Section 6), C–C bond cleavage (Section 7), desorption and catalyst deactivation (Section 8). We examine how these effects collectively determine selectivity, activity and stability. Finally, we discuss how these relationships can guide catalyst design and operating-condition selection and identify the principal challenges for practical application.

## 2. Reaction pathways in GOR

The GOR network comprises coupled electrochemical and chemical steps rather than a single linear sequence (Scheme 1). Glycerol oxidation begins with adsorption on the catalyst surface, and the preferred configuration depends on catalyst composition, surface structure and interfacial environment. Electrochemical dehydrogenation at a terminal or secondary hydroxyl group initially produces GLD or DHA, respectively.^53–56^ Their *ex situ* distributions may not directly reproduce the initial interfacial branching because GLD- and DHA-derived species can undergo further conversion during electrolysis and, after desorption, can interconvert or participate in chemical reactions in the electrolyte.^57,58^ In addition, GLD may exist in hydrated geminal-diol or gem-diolate forms,^59–61^ while complexation with borate species can further affect the stability of C3 intermediates.^62^ Although the principal product branches are supported by GOR experiments, the evidence for individual elementary steps varies and includes *operando* spectroscopy, intermediate and isotopic probes, kinetic analysis and theoretical calculations.

**Scheme 1:**
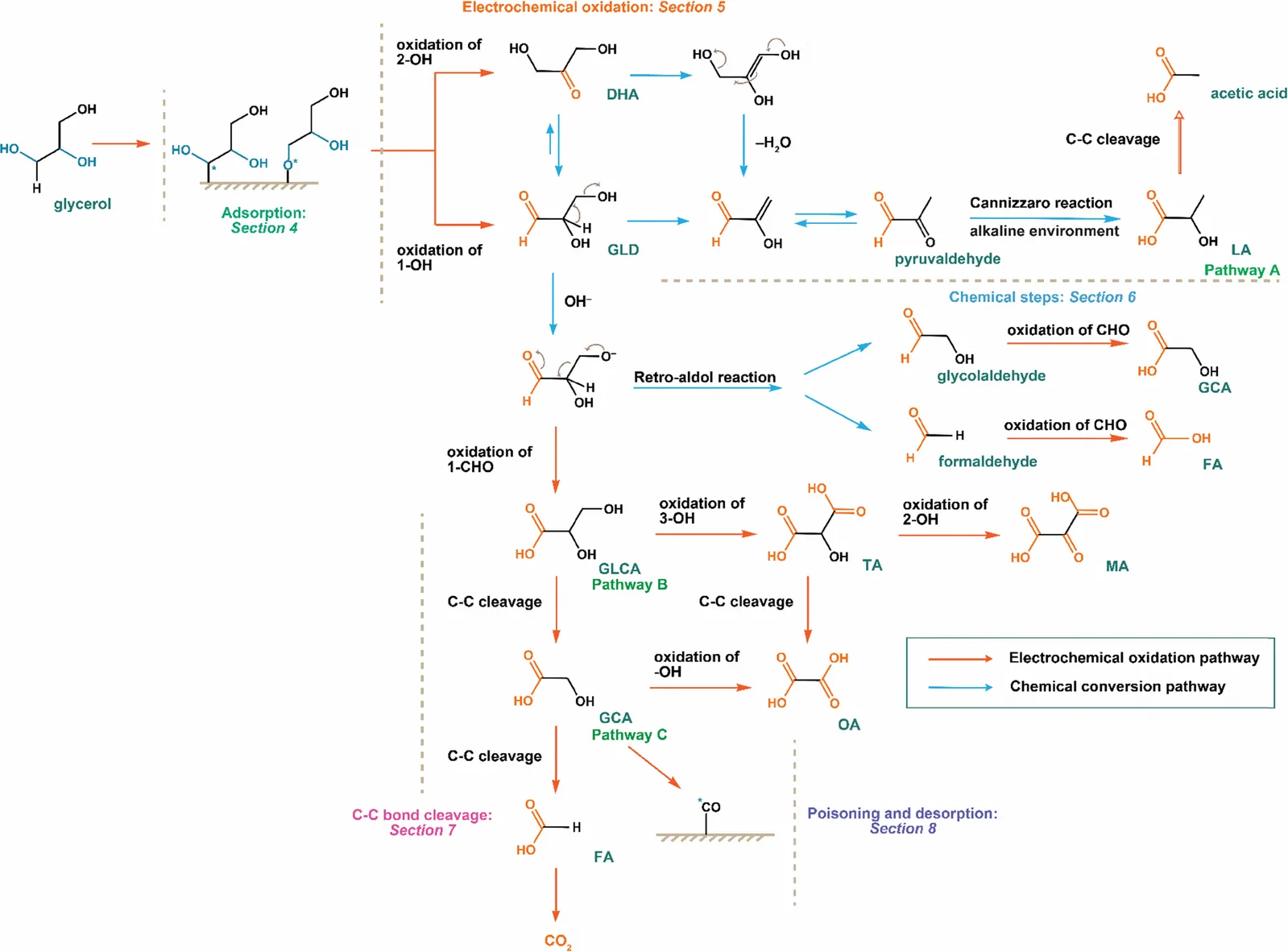
Proposed glycerol oxidation network involving coupled electrochemical (red arrows) and chemical (blue arrows) steps. Adsorption and dehydrogenation initially produce GLD and DHA through oxidation of terminal and secondary hydroxyl groups, respectively. The resulting C3 intermediates can undergo base-promoted transformations leading to LA (pathway A) or consecutive oxidation to GLCA and other C3 products (pathway B). Alternatively, C–C cleavage produces C2/C1 products, including GCA, OA and FA (pathway C), with possible CO_2_ formation under extensive oxidation conditions.

Following the initial oxidation step, subsequent reactions can be grouped into three principal pathways. In pathway A, LA formation is generally attributed to base-promoted chemical transformations of GLD and DHA rather than to a direct electrochemical step.^39,63,64^ In pathway B, consecutive oxidation retains the C3 backbone, converting GLD first to GLCA and then to more highly oxidized C3 products such as TA and MA.^63–65^ Pathway C involves C–C cleavage and produces C2/C1 products, including GCA, OA and FA, with possible CO_2_ formation under extensive oxidation conditions.^66–69^ The competition among these branches determines whether the C3 backbone is retained or progressively fragmented and whether electrochemical or chemical steps dominate the observed product formation.

The three pathways are connected through the adsorption, desorption and consecutive conversion of intermediates. Strongly adsorbed intermediates can remain on the catalyst surface and undergo further electrochemical oxidation, which may increase the probability of C–C cleavage. In contrast, GLD and DHA may desorb into the electrolyte and undergo chemical transformations before further reaction. These intermediates may subsequently be re-adsorbed and undergo additional electrochemical conversion. The balance between these routes depends on intermediate binding strength, interfacial properties, mass transport, applied potential, electrolyte pH and temperature. Applied potential and electrolyte pH are particularly important because they influence the catalyst surface state, oxidation depth and the stability of intermediates in the electrolyte. Higher anodic potentials generally favour deeper oxidation and increase the probability of C–C cleavage.^66–69^ Chemical cleavage of desorbed carbonyl intermediates through a possible retro-aldol transformation has also been proposed, although its contribution under typical electrochemical conditions remains unresolved.^32,68^

The resulting network should therefore be interpreted as a framework that summarizes the principal reaction branches rather than a fixed sequence followed by every catalyst. This framework provides a common basis for comparing how changes in the catalytic environment shift the balance among these pathways. Section 3 evaluates the types and limitations of evidence used to identify these pathways, whereas Sections 4–8 discuss how the catalytic environment regulates individual reaction steps.

## 3. Approaches to probe the reaction pathway

The coupled electrochemical and chemical pathways involved in GOR cannot be resolved using a single method because many intermediates are transient and catalyst surface states evolve with applied potential. Theoretical calculations identify energetically plausible elementary steps and catalyst descriptors, whereas *in situ* and *operando* spectroscopy tracks intermediates and catalyst states under reaction conditions. Electrolysis of proposed intermediates, comparative reactant studies and isotopic labelling provide complementary evidence for pathway sequence and carbon fate.^69–73^ These approaches are therefore evaluated according to the mechanistic evidence they provide and their respective limitations. Detailed catalyst-specific interpretations are discussed in Sections 4–8.

### 3.1. Insights into GOR pathways from quantum chemistry calculations

Density functional theory (DFT) provides atomic-scale information on adsorption configurations, bond-activation barriers, reaction energetics and transition states. It can compare competing elementary steps and identify descriptors of activity or pathway preference, although its conclusions depend on the surface structures and electrochemical environment represented in the model. Defects, interfaces and reconstructed working surfaces remain challenging to describe.^74–78^ For example, calculations on Pt(111) compare competing C–C and C–O cleavage pathways,^79^ while selectivity maps based on carbon- and oxygen-binding energies distinguish preferences for the first two glycerol dehydrogenation steps (Fig. 2a).^80^ Such maps provide a basis for comparing model surfaces, but do not independently establish the product distribution on a working catalyst.

**Fig. 2:**
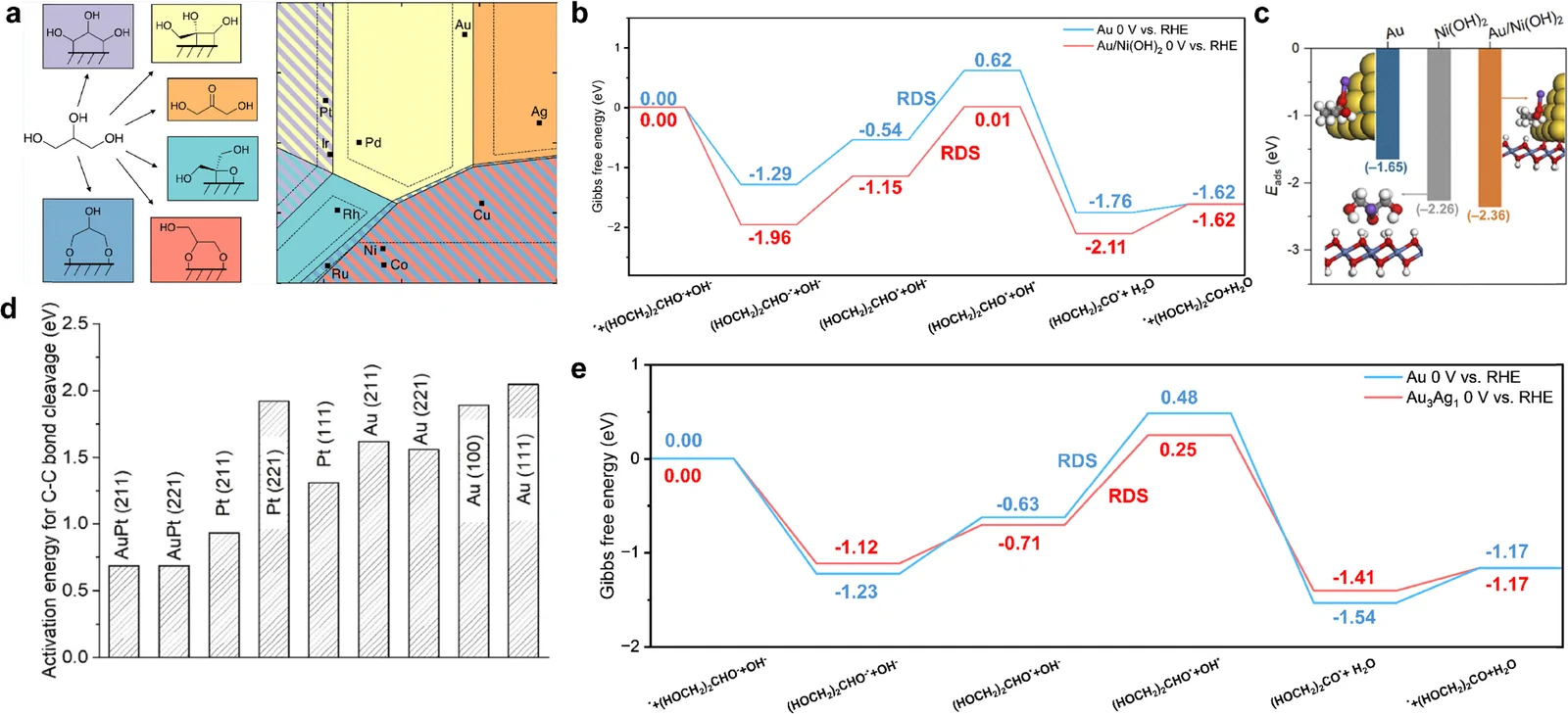
(a) Theoretical selectivity map predicting the preferred dehydrogenation pathway based on C and O binding energies. Reproduced from Valter *et al.*^80^ under the terms of the Creative Commons Attribution 4.0 International License (CC BY 4.0). Copyright 2021 The Authors, published by American Chemical Society. (b) Gibbs free energy landscape for electrocatalytic oxidation of glycerol alkoxide to DHA over Au/Ni(OH)_2_ and Au at 0 V *vs.* RHE. Adapted with permission from Yan *et al.*^81^ Copyright 2023, American Chemical Society. (c) Adsorption energies of GLY in the form of alkoxide on Au, Ni(OH)_2_, and Au/Ni(OH)_2_, respectively. The optimized geometries of (HOCH_2_)_2_CHO* for Au, Ni(OH)_2_, and Au/Ni(OH)_2_ are displayed. Reproduced with permission from Yan *et al.*^81^ Copyright 2023, American Chemical Society. (d) DFT calculations on relative energy of C–C bond cleavage on AuPt(211), AuPt(221), Pt(211), Pt(221), Pt(111), Au(211), Au(221), Au(100), and Au(111) facets. Reproduced with permission from Wei *et al.*^84^ Copyright 2023, American Chemical Society. (e) Gibbs free energy landscape for electrocatalytic oxidation of glycerol alkoxide to DHA over the Au(111) and Au_3_Ag(111) surfaces at 0 V *vs.* RHE. Adapted with permission from Yan *et al.*^42^ Copyright 2024, Wiley-VCH GmbH.

DFT can also distinguish interfacial adsorption effects from changes in the energetics of individual elementary steps. At Au/Ni(OH)_2_ interfaces, calculations indicate stronger glycerol-alkoxide adsorption through combined Au–alkoxide binding and hydrogen bonding, whereas OH* formation remains the step identified as rate determining in the reported analysis (Fig. 2b and c).^81^ MoO_*x*_/Pt also similarly exhibits a stronger calculated affinity for glycerol than Pt.^82^ Electronic modification provides a contrasting example: calculations on Au–Ag indicate more favourable OH* formation than on Au (Fig. 2e), supporting a contribution beyond enhanced reactant adsorption.^42,83^ These examples illustrate different contributions to enhanced activity: increasing interfacial reactant availability and facilitating formation of a reactive surface species. Stronger adsorption alone does not demonstrate a lower activation barrier for subsequent oxidation. The corresponding adsorption and dehydrogenation mechanisms are discussed in Sections 4 and 5.

Local site geometry can also be tested computationally. Stepped Pt–Au models predict more favourable C–C cleavage than several terrace models (Fig. 2d).^84^ However, the associated experimental selectivity measurements concerned ethanol oxidation. This example therefore supports a structural hypothesis for alcohol oxidation rather than directly establishing the same site-specific mechanism during GOR.

Accounting for potential and structural heterogeneity extends these comparisons towards operating conditions. Grand canonical DFT (GC-DFT) can assess potential-dependent thermodynamics and the effects of oxygen coverage,^39,85^ but a predicted thermodynamic preference does not establish an experimentally validated switch between C3 retention and C–C cleavage. For multielement catalysts, machine-learning-assisted Monte Carlo sampling has been combined with DFT to compare intermediate adsorption across CoNiCuMnMo nanoparticle configurations.^86^ The resulting activity trends were consistent with electrochemical measurements, although this agreement did not directly identify the proposed active sites under operating conditions.

DFT is therefore most useful here for separating possible contributions of adsorption, site geometry and elementary-step energetics. Its principal limitation is the representation of heterogeneous, adsorbate-covered and evolving surfaces. Agreement with measured activity or selectivity supports the consistency of a proposed mechanism, but spectroscopic and reaction-probe evidence is needed to distinguish it from alternative explanations (Sections 3.2 and 3.3).

### 3.2. *Operando* spectroscopic identification of electrochemical GOR pathway

*In situ* and *operando* measurements test whether proposed intermediates and catalyst states occur during reaction. Vibrational methods, including attenuated total reflection Fourier-transform infrared spectroscopy (ATR-FTIR), surface-enhanced infrared absorption spectroscopy (SEIRAS), and surface-enhanced Raman spectroscopy (SERS),^24,50,87–89^ detect molecular and adsorbed species, while differential electrochemical mass spectrometry (DEMS) follows gaseous products.^90^*Operando* X-ray absorption spectroscopy (XAS) provides complementary information on oxidation state and local coordination.^47,84,91^ Their combined value lies in connecting changes in surface chemistry with changes in reaction products.

Fig. 3 and Table 1 compile representative vibrational assignments together with the catalysts, electrolytes and measurement conditions from which they were obtained.^92–95^ These bands provide a useful basis for connecting spectral signatures with oxidation depth. Alcohol-derived C–O vibrations mainly reflect glycerol or alkoxide-like species, carbonyl-related bands indicate C3 intermediates such as GLD and DHA, carboxylate/formate bands point to further oxidation products, and CO-related bands are associated with strong adsorption, poisoning, and deep oxidation. These assignments are reference ranges rather than universal fingerprints: overlapping bands, solvent absorption and differences between adsorbed and dissolved species can complicate identification. Measurements in D_2_O, for example, have helped resolve DHA-related signals obscured in aqueous spectra.^96^ Assignment is therefore stronger when spectral changes are supported by reference compounds, potential or time dependence, and independent product analysis. Nevertheless, they help translate spectral evolution into mechanistic information on whether GOR remains within C3-preserving pathways or proceeds towards C–C cleavage and C1/C2 products.

**Fig. 3:**
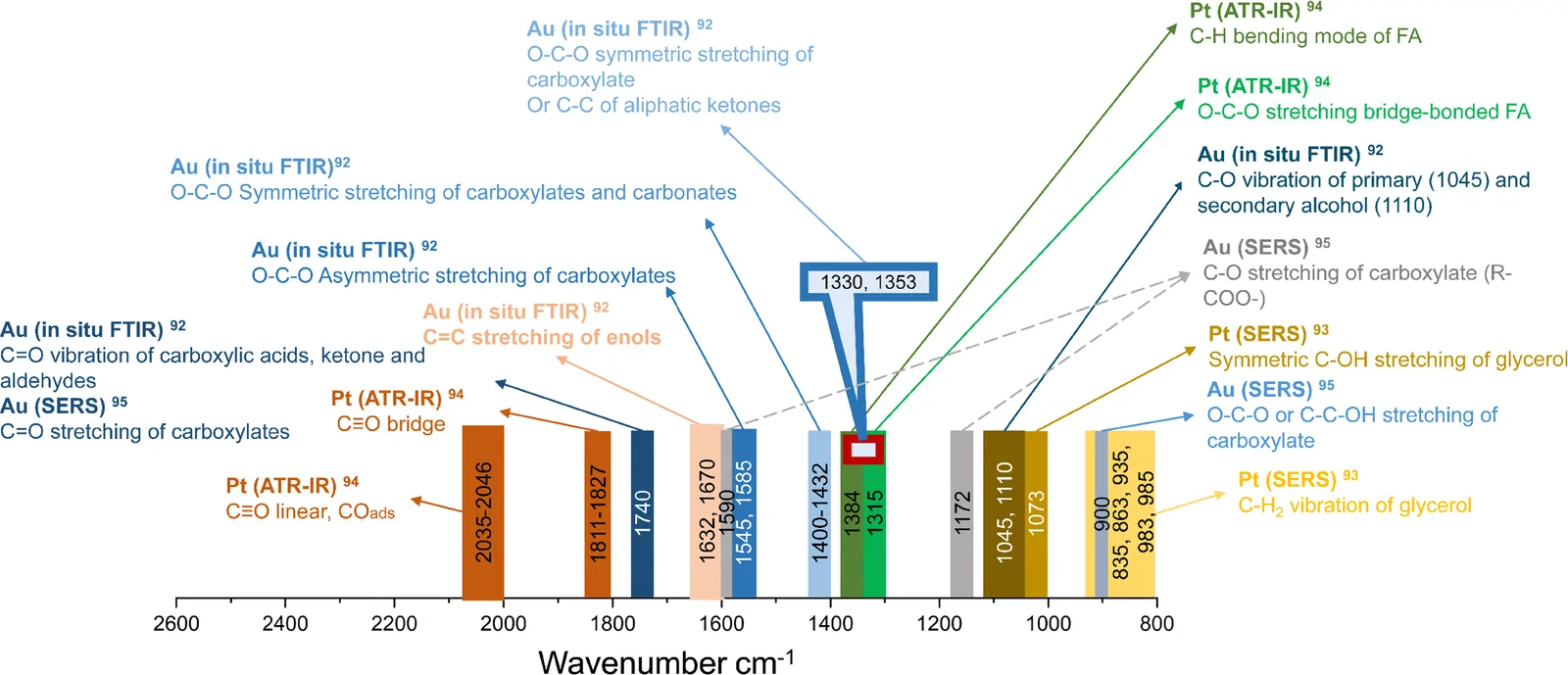
Representative vibrational bands reported in the literature for glycerol electrooxidation-related species and selected reference intermediates/products.^92–95^ Accordingly, this figure should be regarded as a literature-based summary of characteristic bands rather than a dataset obtained from a single technique or catalyst system.

**Table 1:** Representative vibrational band assignments and measurement conditions

Technique	Peak (cm^−1^)	Assignment	Catalyst	Electrolyte	Potential	Ref.
*In situ* FTIR	1045, 1110	C–O vibration of primary (1045) and secondary alcohol (1110)	Au/C	0.1 M NaOH + 0.1 M glycerol	Spectra at 0.05 V, then 0.1–1.6 V *vs.* RHE in 0.1 V steps. Reference spectrum at 0.05 V *vs.* RHE	92
	1330, 1353	O–C–O symmetric stretching of carboxylate or C–C of aliphatic ketones				
	1400, 1424, 1432	O–C–O Symmetric stretching of carboxylates and carbonates				
	1545, 1585	O–C–O Asymmetric stretching of carboxylates				
	1632, 1670	C=C stretching of enols				
	1740	C=O vibration of carboxylic acids, ketone and aldehydes				
SERS	835, 863, 935, 983, 985	C–H_2_ vibration of glycerol	Pt/C	0.3 M KOH + 1.5 M glycerol	CV, −0.6 to 0.4 V *vs.* NHE at 1 mV s^−1^, four cycles	93
	1073, 1124	Symmetric C–OH stretching of glycerol				
ATR-IR	1315	O–C–O stretching of bridge-bonded FA	Pt/C (60 wt% Pt on carbon)	0.5 M HClO_4_ + 25 mM HCOOH	Cyclic voltammogram from 0.0 V to 1.2 V *vs.* RHE, scan rate 1 mV s^−1^	94
	1384	C–H bending mode of FA				
	1811–1827	C–O stretch of bridging adsorbed CO			Background at 0 V before addition of HCOOH	
	2035–2046	C–O stretching of linearly adsorbed CO				
SERS	900	O–C–O or C–C–OH stretching of carboxylate	Au nanoshells	1.3 mM glycerol in water, at pH 11	N/A	95
	1172, 1590	C–O stretching of carboxylate (R-COO^−^)				
	1740	C=O stretching of carboxylates				

Two examples illustrate how complementary measurements constrain pathway interpretation. On Pt in acidic electrolyte, simultaneous ATR-FTIR and DEMS measurements detected adsorbed CO and CO_2_ evolution, connecting surface carbonaceous species with C–C cleavage and deeper oxidation (Fig. 4a and b).^97^ On Ni catalysts, time-resolved Raman signals assigned to DHA and GLCA first increased and then declined, while signals associated with C2 species increased. Together with potential-dependent FTIR detection of FA, these observations support consecutive conversion of initially formed C3 intermediates (Fig. 4c and d).^70^

**Fig. 4:**
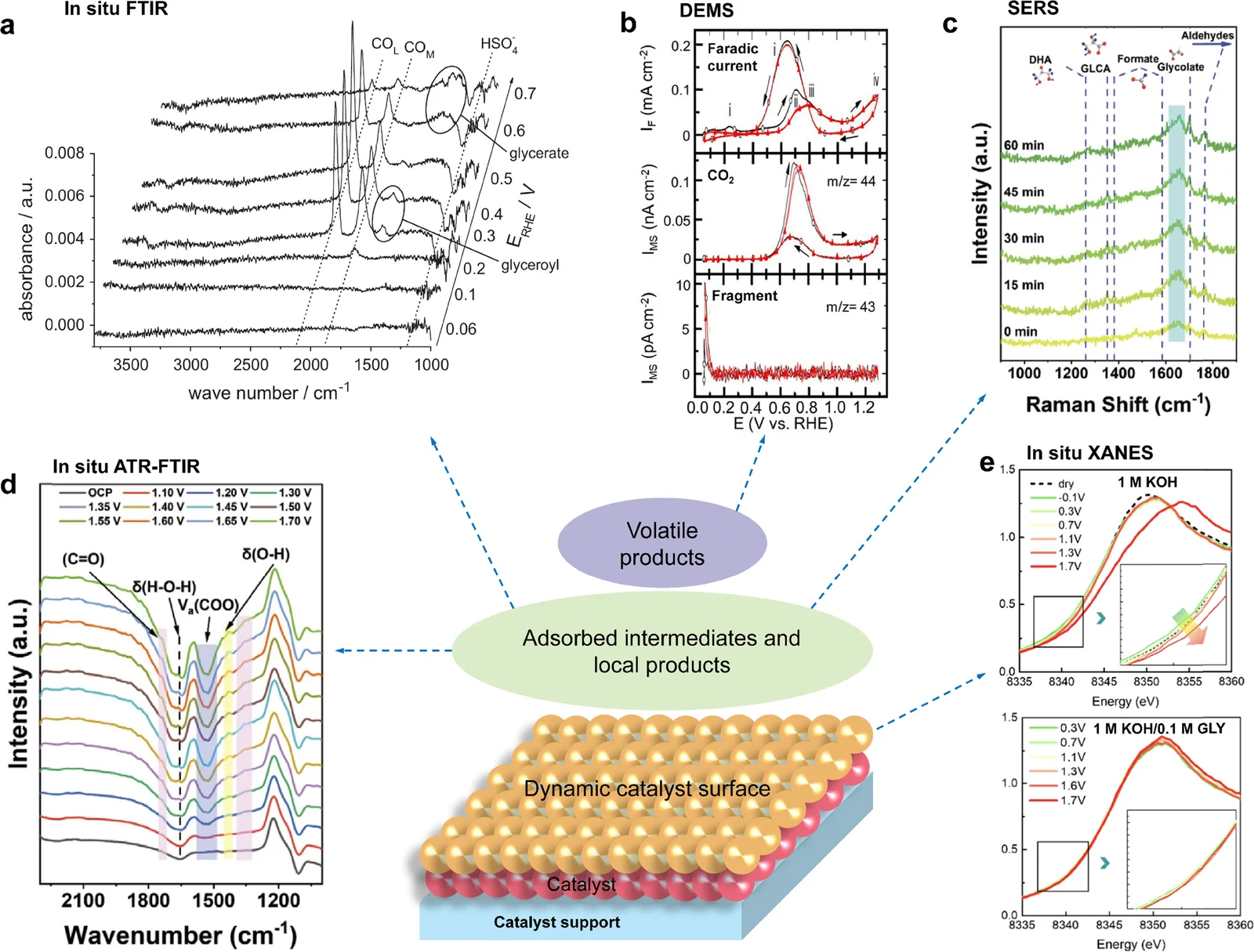
Dynamic processes in electrochemical GOR from *in situ* and *operando* techniques. (a) ATR-FTIR spectra recorded about 3-4 s after electrolyte exchange from 0.5 M H_2_SO_4_ to 0.1 M GLY containing 0.5 M H_2_SO_4_ at different potentials. Reproduced with permission from Schnaidt *et al.*^97^ Copyright 2011, Elsevier. (b) Potentiodynamic electrooxidation of glycerol: faradaic current; mass spectrometric current for *m*/*z* = 44, *m*/*z* = 43. Reproduced with permission from Schnaidt *et al.*^97^ Copyright 2011, Elsevier. (c) *in situ* Raman spectra of Ni catalysts at a constant potential of 1.35 V (*vs.* RHE) for 60 min during GOR. Reproduced with permission from Wang *et al.*^70^ Copyright 2024, Wiley-VCH GmbH. (d) *In situ* ATR-FTIR spectra of Ni catalysts for GOR collected at different potentials from open circuit potential to 1.7 V *vs.* RHE. Reproduced with permission from Wang *et al.*^70^ Copyright 2024, Wiley-VCH GmbH. (e) *In situ* XANES spectra at Ni K-edge for PtNi_2_ in 1 M KOH and 1 M KOH/0.1 M glycerol. Reproduced from Luo *et al.*^91^ under the terms of the Creative Commons Attribution 4.0 International License (CC BY 4.0). Copyright 2022 The Authors, published by American Chemical Society. The central schematic represents a dynamic catalyst surface during GOR. Representative spectra are compiled from different literature studies and are shown to illustrate technique-specific mechanistic insights rather than direct comparison under a single catalytic system.

XAS addresses a different question: whether the catalyst state used to interpret the reaction persists in the reacting electrolyte. For PtNi catalysts, the Ni K-edge changed under anodic polarization in KOH, whereas the spectra remained nearly unchanged in the presence of glycerol (Fig. 4e).^91^ This contrast shows that the reaction environment affects Ni oxidation and constrains descriptions based on the glycerol-free surface. The interpretation of Ni-derived sites and their electronic influence on Pt is considered in Section 5.1; the XAS observation alone does not uniquely establish that mechanism.

Together, *in situ* and *operando* measurements provide complementary evidence for intermediate conversion and catalyst-state evolution. Mechanistic assignments are strongest when changes in intermediate signals, product formation and catalyst state are correlated under comparable reaction conditions.

### 3.3. Unravelling reaction pathways through intermediate and isotopic probes

Intermediate electrolysis, comparative reactant studies and isotopic labelling constrain different parts of the reaction network. Matched non-electrolysed controls are particularly useful for distinguishing accessible electrochemical conversions from chemical reactions that occur without electrode-driven electron transfer.

LA formation during DHA or GLD electrolysis is comparable to that observed in non-electrolysed control experiments (Fig. 5a), indicating that chemical transformations in the alkaline electrolyte contribute substantially to LA formation.^67^ The detailed chemical pathway is discussed in Section 6. Conversely, formation of GCA, OA and FA during GLCA electrolysis supports its possible involvement in consecutive oxidation and C–C cleavage.^67,98,99^ Such tests establish accessible conversions, although their contribution during glycerol electrolysis must still be assessed under the relevant reaction conditions.

**Fig. 5:**
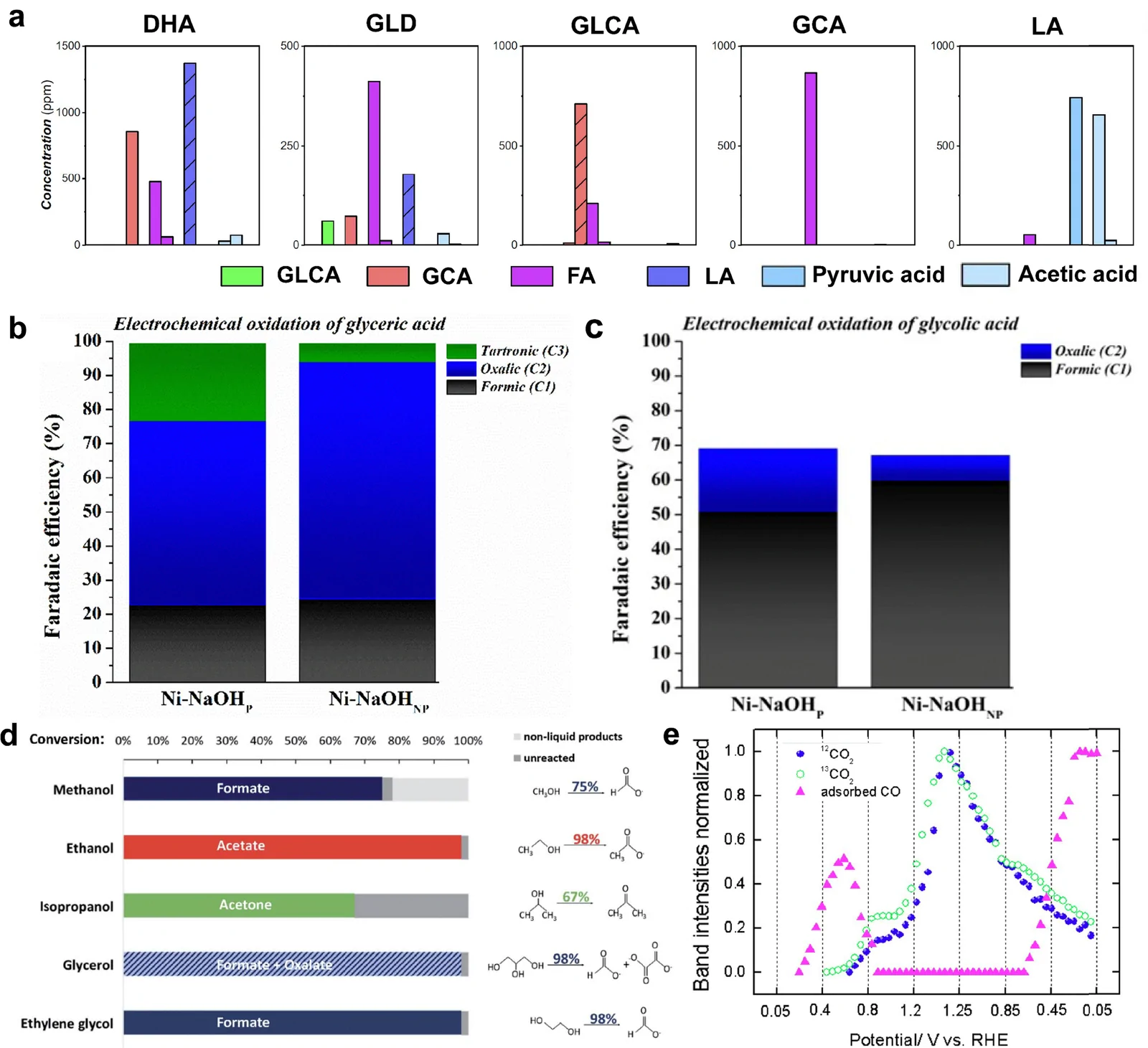
(a) Anolyte composition (in ppm) of the divided cell electrolysis of 15 mL of KOH 1 M and DHA 0.1 M, GLD 0.01 M, GLCA 0.1 M, GCA 0.1 M, and LA 0.1 M. Reproduced with permission from Molera *et al.*^67^ Copyright 2025 The Authors. ChemSusChem published by Wiley-VCH GmbH. faradaic efficiencies (FE%) to liquid products identified and quantified by HPLC, using the Ni-NaOH_P_ (purified NaOH electrolyte) and Ni-NaOH_NP_ (non-purified NaOH electrolyte) electrodes, for the electrochemical oxidation of (b) GLCA, and (c) GCA. Reproduced from Santana *et al.*^99^ under the terms of the Creative Commons Attribution 4.0 International License (CC BY 4.0). Copyright 2023 The Authors. ChemElectroChem published by Wiley-VCH GmbH. (d) Summary of alcohol oxidation processes catalysed by Ni_12_P_5_ and their various value-added oxidation products. Reproduced from ref. 100 with permission from the Royal Society of Chemistry. (e) Band intensities as a function of labelled glycerol + 0.1 mol dm^−3^ HClO_4_ on Pt/C nanoparticles. Reproduced with permission from Fernández *et al.*^103^ Copyright 2013, Elsevier.

Control of the electrolyte is essential to these comparisons. Fe impurities accelerated C–C cleavage during intermediate oxidation on Ni catalysts, altering the resulting C1/C2 distribution (Fig. 5b and c).^99^ These results also demonstrate that electrolyte impurities can influence conclusions drawn from intermediate electrolysis. Comparative reactant studies provide another constraint: on nickel phosphide catalysts, glycerol and ethylene glycol underwent C–C cleavage, whereas ethanol and isopropanol did not under comparable conditions (Fig. 5d).^100^ This comparison implicates vicinal hydroxyl groups in the reported reactivity. Methanol also formed FA, but without C–C cleavage, illustrating why product identity must be interpreted together with substrate structure.

Isotopic labelling traces carbon origins more directly. *In situ* FTIR measurements with terminally labelled glycerol (^13^CH_2_OH–^12^CHOH–^13^CH_2_OH) detected CO_2_ derived from both terminal and central carbon atoms on Pt, showing that cleavage was not restricted to one carbon position.^101–103^ Potential-dependent signals also distinguished CO-mediated oxidation from pathways associated with surface Pt–O species (Fig. 5e).^102,103^ Fully ^13^C-labelled glycerol has additionally been used to separate glycerol-derived CO_2_ from oxidation of pre-adsorbed CO, providing evidence for competitive site blocking.^104^

These approaches constrain reaction sequence, chemical contributions and carbon fate from different directions. Combined with calculations and *operando* measurements, they provide the evidence base for examining how catalytic environments regulate each step in Scheme 1.

## 4. Adsorption as the origin of pathway selectivity in GOR

Within the mechanistic framework shown in Scheme 1, adsorption is the entry step preceding the formation of GLD and DHA. It establishes whether glycerol-derived species preferentially enter C3-preserving pathways or pathways involving C–C cleavage. Section 4 examines how metal identity, surface ensemble geometry, electronic modulation and catalyst–support interfaces regulate this initial adsorption step. The discussion therefore considers the preferred C- or O-bound configurations, adsorption energetics, the number of metal–carbon contacts, interfacial reactant coverage and adsorption kinetics. Together, these structural, energetic and interfacial aspects provide a basis for interpreting differences in activity and C3 *versus* C2/C1 product distributions among metals, alloys and supported catalysts. Adsorption therefore influences the surface conditions for subsequent dehydrogenation and pathway branching.

### 4.1. Intrinsic adsorption modes on metal surfaces

#### 4.1.1. Metal-dependent adsorption mode

The adsorption mode of glycerol is fundamentally governed by the adsorption free energy on the metal surface, which directly reflects the metal-adsorbate interaction and the coordination/electronic environment of surface sites. This energetic preference dictates the dominant anchoring atom and thus separates glycerol adsorption into two regimes: carbon-bound (C–M) *versus* oxygen-bound (O–M) adsorption.^105–111^

Calculations comparing C- and O-bound species derived from initially neutral glycerol show that Pt(111) and Pd(111) favour carbon anchoring, whereas Ag(111), Cu(111) and Ru(111) favour oxygen anchoring (Fig. 6a and b).^112^ On Pt(111), the reported free energies for the initial C- and O-bound configurations are approximately 0.37 and 0.75 eV, respectively. Once GLD forms, adsorption through its aldehyde carbon is more favourable, with a reported adsorption free energy of −0.30 eV. On Ag(111), the corresponding comparison instead favours O-bound glycerol-derived species. These results illustrate how the same reactant and intermediate can experience different stabilisation on different surfaces. Au highlights why the identity of the adsorbing species matters. The neutral glycerol derived model shows similar stability for C- and O-bound configurations on Au(111),^112^ whereas alkaline deprotonation produces alkoxide species that Au can stabilise through Au–O interactions.^81,113^ Thus, a surface preference inferred from one model cannot be transferred directly to a different electrolyte or reactant speciation. Glycerol deprotonation and OH-assisted activation can change the adsorbing species and the relative rates of competing initial pathways.^39^ Water influences solvation and proton transfer, while electrolyte cations can alter the formation of OH* and oxide species.^80,114^

**Fig. 6:**
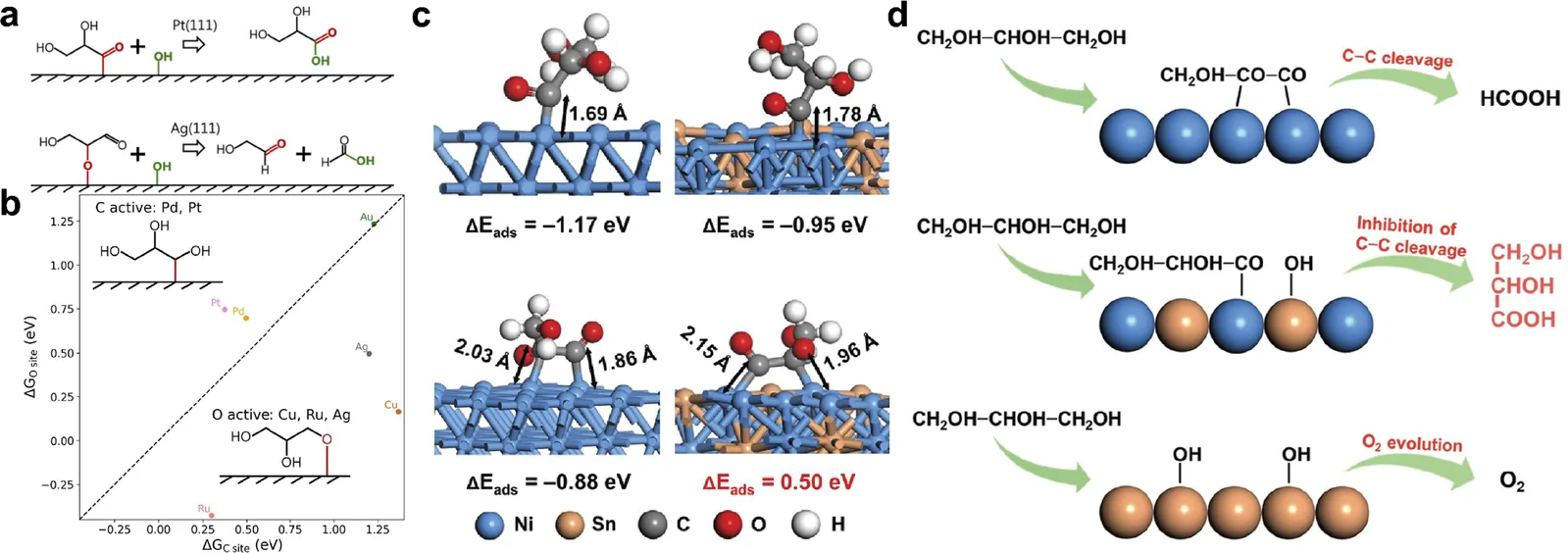
(a) Comparison of adsorption configurations and reaction pathways of glycerol-derived intermediates on Pt(111) and Ag(111) surfaces. Reproduced with permission from Meng *et al.*^112^ Copyright 2024, American Chemical Society. (b) Gibbs free energy difference for glycerol adsorption *via* C* adsorption (Δ*G*_C site_) *versus* O* adsorption (Δ*G*_O site_) on (111) metal surfaces. Reproduced with permission from Meng *et al.*^112^ Copyright 2024, American Chemical Society. (c) Adsorption configurations, adsorption energies, and bond lengths at Ni(111) and Ni_3_Sn(201) with mono-carbon binding intermediate (CH_2_OH–CHOH–*CO) and multi-carbon binding intermediate (CH_2_OH–*CO–*CO). Reproduced with permission from Lyu *et al.*^24^ Copyright 2024, Wiley-VCH GmbH. (d) Schematic illustrations of GOR on different catalysts, top panel: Ni, middle panel: Ni–Sn alloy, bottom panel: Sn. Reproduced with permission from Lyu *et al.*^24^ Copyright 2024, Wiley-VCH GmbH.

Descriptor calculations express these differences through the binding energies of CH_3_O* and *CH_2_OH, representing oxygen and carbon affinity, respectively.^72,73,80,115^ Their difference provides a relative ordering of the modelled metals from oxygen-preferring to carbon-preferring behaviour, while the thermodynamic selectivity maps retain both energies as independent variables.^80^ These descriptors rationalise initial dehydrogenation preferences, but their quantitative relationship with final product selectivity across metals and alloys remains unestablished. They are therefore best used to formulate adsorption hypotheses that can be assessed against experimental evidence.

Composition can regulate not only the preferred anchoring atom but also the relative stability of adsorption configurations involving different numbers of carbon–metal contacts. In ordered Ni_3_Sn, DFT calculations suggest that Sn incorporation weakens Ni–C interactions and preferentially destabilises intermediates with multiple carbon–metal contacts, favouring predominantly single-carbon binding (Fig. 6c).^24^ This interpretation is consistent with suppressed C–C cleavage and enhanced GLCA selectivity relative to Ni (Fig. 6d). However, the proposed adsorption configurations have not been directly identified under *operando* GOR conditions.

Together, these comparisons show how catalyst composition influences the preferred anchoring atom and the relative stability of configurations with different carbon-metal contacts, with these preferences depending on the adsorbing species and reaction conditions. For C3-targeted oxidation, they suggest maintaining sufficient adsorption for activation while limiting the stabilisation of configurations associated with carbon-skeleton fragmentation.

#### 4.1.2. Adsorption configurations, ensemble effects and electronic effects

The anchoring atom is only one aspect of adsorption. Glycerol-derived species may also bind through different numbers of carbon atoms, depending on the availability of adjacent metal sites. Multiple surface contacts can stabilise dissociative configurations, whereas restricting these contacts can favour pathways that retain the C3 backbone. Site geometry therefore provides a second means of controlling adsorption without requiring a complete change from carbon to oxygen anchoring.

Restricting adjacent Pt sites can suppress adsorption configurations involving multiple carbon–metal contacts. Glycerol-derived species have been proposed to bind Pt through one, two or three carbon atoms, with neighbouring Pt sites accommodating multiply bound intermediates that more readily undergo fragmentation to CO and subsequent oxidation products.^116^ Bi adatoms reduce the availability of these site combinations through a steric/site-blocking effect. The resulting suppression of multiply bound species was correlated with increased GLCA formation relative to unmodified Pt (Fig. 7a), linking ensemble restriction to enhanced C3 retention. PtAu alloying similarly reduces contiguous Pt ensembles and was interpreted to favour binding through a single terminal carbon, suppressing configurations associated with C–C cleavage and rapid deactivation (Fig. 7b).^117^ These systems support the same geometric principle through different modifications: limiting the combinations of adjacent sites available to a glycerol-derived intermediate.

**Fig. 7:**
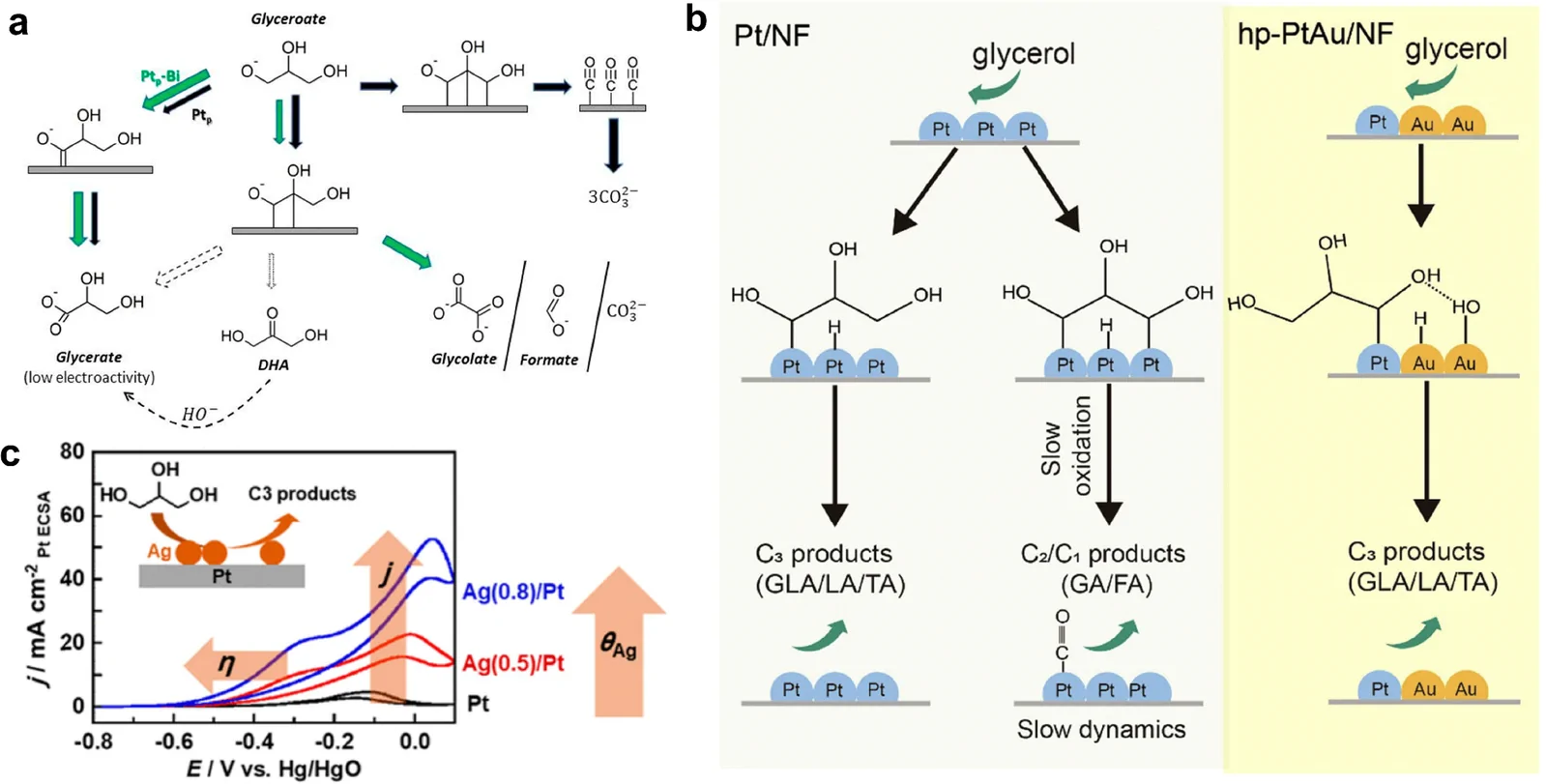
(a) Reaction pathways for the GOR on Pt_p_ and Pt_p_-Bi. Reproduced with permission from de Souza *et al.*^116^ Copyright 2019, American Chemical Society. (b) Schematic diagrams of the adsorption configurations of glycerol molecules on hp-PtAu/NF and Pt/NF surfaces. Abbreviations are retained from the original figures: GLA and GA correspond to GLCA and GCA, respectively, in the main text. Reproduced from ref. 117 with permission from the Royal Society of Chemistry. (c) Effect of Ag modification on the glycerol electrooxidation activity of Pt catalysts. Reproduced with permission from Hamada *et al.*^118^ Copyright 2024, American Chemical Society.

Beyond geometric adsorption configurations, adsorption behaviour can also be regulated through electronic effects that modify the strength and kinetics of glycerol-derived intermediates on metal surfaces. Ag incorporation into Pt altered glycerol adsorption behaviour and accelerated the formation of adsorbed species at low potentials (Fig. 7c).^118^ Similarly, Ni in PtNi catalysts was proposed to act mainly through electronic modulation of Pt, tuning CO*/OH* binding to improve activity and poisoning tolerance, because glycerol adsorption restricts hydroxyl activation on Ni-derived sites.^91^

Together, these examples distinguish two complementary contributions: ensemble regulation changes which adsorption configurations are accessible, whereas electronic modulation alters the relative binding strengths of glycerol-derived intermediates, CO* and OH*. These effects can coexist, jointly influencing adsorption geometry and binding strength and thereby contributing to glycerol activation and subsequent pathway selection.

### 4.2. Interfacial regulation of adsorption

Beyond the intrinsic adsorption characteristics of noble metals, catalyst–support interfaces play a critical role in regulating glycerol adsorption during electrooxidation. In many effective systems, enhanced glycerol adsorption is associated with interfaces that contain surface hydroxyl groups or oxygen-containing functionalities, which can directly interact with glycerol molecules through hydrogen bonding or Lewis acid–base interactions. Rather than acting as inert scaffolds, functionalized supports and secondary domains reshape the local adsorption environment by increasing reactant coverage and modifying adsorption kinetics at interfacial sites. As a result, the interface provides an additional degree of freedom for tuning the early stages of glycerol electrooxidation and ultimately the accessibility of distinct reaction pathways.

A primary function of the interface is to enhance the local concentration of glycerol-derived species near metal active sites. For noble metals with weak intrinsic affinity towards polyols, such as Au, adsorption can become kinetically limited by low surface coverage. At hydroxyl-rich Ni(OH)_2_/Au interfaces, hydrogen bonding between surface hydroxyl groups and glycerol hydroxyls increases the local concentration of glycerol in the vicinity of Au nanoparticles (Fig. 8a).^81^ This support-assisted enrichment improves interfacial reactant coverage without substantially strengthening metal–carbon interactions, thereby enhancing activity while preserving the tendency towards C3-selective pathways.

**Fig. 8:**
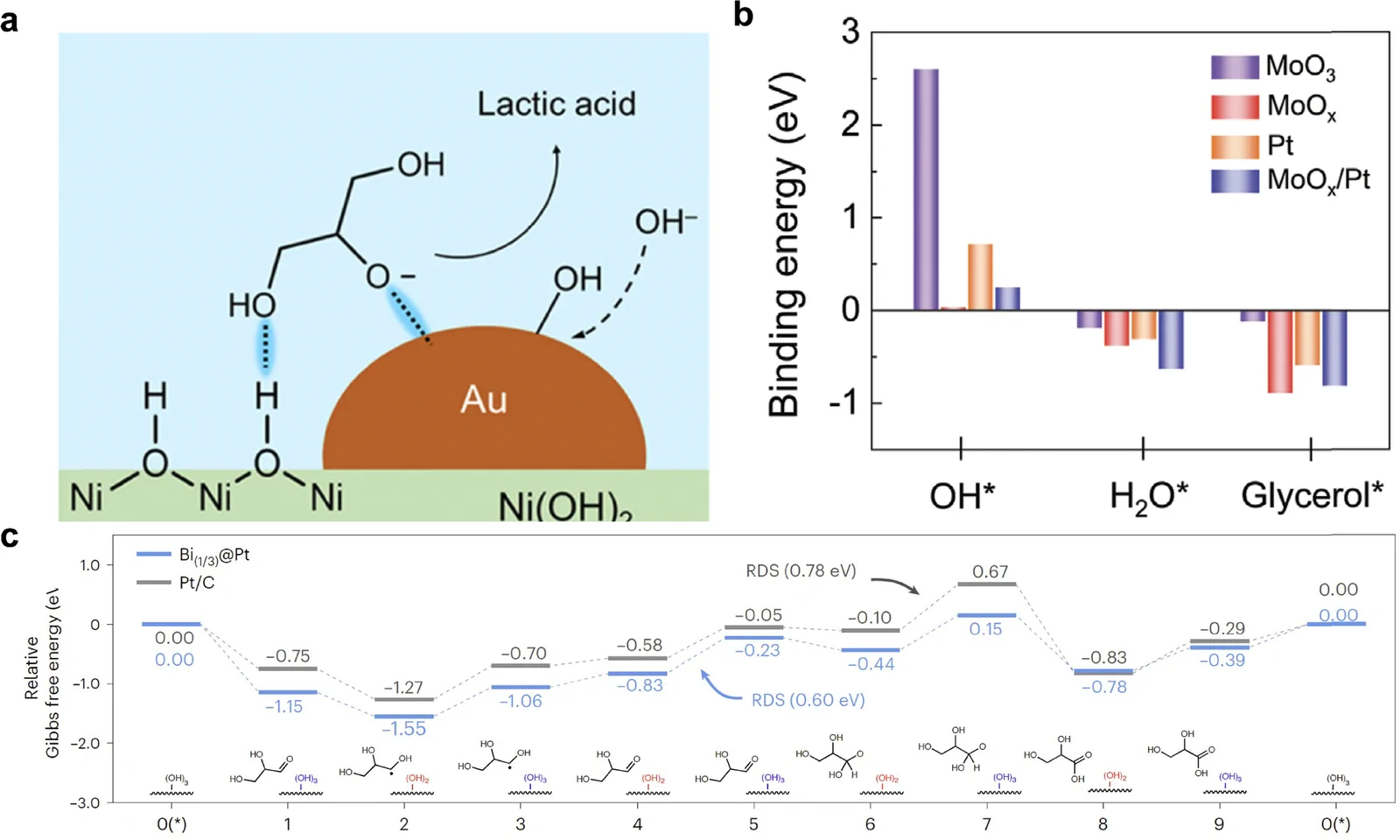
(a) Schematic illustration of the adsorption configuration of glycerol alkoxide at the Au/Ni(OH)_2_ interface. Reproduced with permission from Yan *et al.*^81^ Copyright 2023, American Chemical Society. (b) Calculated binding energy of OH, H_2_O, and glycerol on different catalyst surfaces. Reproduced from Yu *et al.*^82^ under the terms of the Creative Commons Attribution 4.0 International License (CC BY 4.0). Copyright 2021 The Authors, Small published by Wiley-VCH GmbH. (c) DFT calculation of reaction pathway on Bi_(1/3)_@Pt and Pt/C. Reproduced with permission from Oh *et al.*^120^ Copyright 2025 The Authors, under exclusive licence to Springer Nature Limited.

Similar interfacial enrichment effects have been reported in oxide-supported catalysts.^119^ At oxygen-vacancy-rich MoO_*x*_/Pt interfaces, MoO_*x*_ domains adsorb glycerol molecules and facilitate their delivery to adjacent Pt sites (Fig. 8b),^82^ effectively lowering the kinetic barrier associated with the initial adsorption step, leading to improved catalytic performance. Surface modification with hydroxylated species can also regulate glycerol adsorption. In Bi_(1/3)_@Pt, DFT calculations using a Bi(OH)_3_-modified Pt(111) model indicate that the initial adsorption of glycerol is more favourable than on pristine Pt(111) (Fig. 8c).^120^ This comparison illustrates how hydroxylated surface modifiers can alter the adsorption energetics of glycerol at Pt-based interfaces.

Although the specific interfacial interactions differ, these interfacial modifications share two coupled effects: enhancing local reactant coverage and reshaping adsorption kinetics at interfacial sites. By simultaneously facilitating glycerol activation and surface regeneration, interfaces bridge intrinsic metal adsorption properties and reaction pathway selection, providing a mechanistic basis for controlling the depth and selectivity of glycerol oxidation through interface engineering.

In summary, glycerol adsorption is governed by a hierarchy of intrinsic metal affinity, surface ensemble structure, electronic effects, and interfacial microenvironment. These factors do not act independently. Metal identity determines whether glycerol-derived species preferentially bind through carbon or oxygen atoms. Surface ensembles determine whether the molecule can form single- or multi-carbon contacts. Electronic effects tune the relative stability of glycerol-derived fragments, CO*, and OH*. Interfaces regulate local reactant coverage and adsorption kinetics. Together, these variables define whether adsorption initiates a C3-preserving pathway or a deeper oxidation route involving C–C cleavage.

The product distributions summarized in Fig. 9 provide a qualitative link between adsorption behaviour and pathway selectivity. For noble-metal-based catalysts, C3 products dominate in many reported systems, but the identity of the C3 product varies substantially. Au- and Au-based systems often show high LA or GLCA fractions, consistent with oxygen-bound or alkoxide-like adsorption that preserves the carbon backbone while allowing subsequent C3 chemical transformation. Pt- and Pd-based systems more frequently show substantial GLCA and TA formation, indicating that these surfaces can promote consecutive oxidation of C3 intermediates without necessarily causing complete C–C cleavage. However, when strong C-bound adsorption or contiguous Pt/Pd ensembles favour multi-carbon anchoring, C2/C1 products such as GCA and FA become more pronounced.

**Fig. 9:**
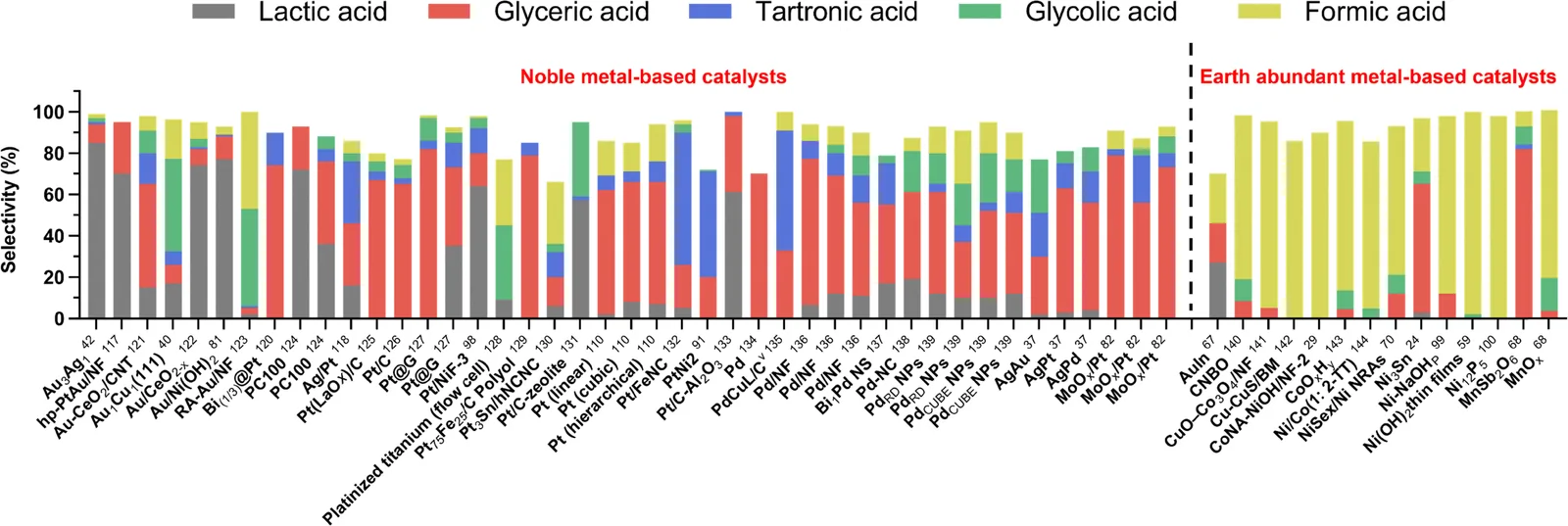
Reported product distributions in glycerol electrooxidation over different catalytic systems. The numbers shown in figure correspond to the literature references from which the data were extracted. The data were obtained under different experimental conditions, including different catalyst loadings, supports, and electrolytes, and are therefore shown to illustrate general selectivity trends rather than enable direct quantitative comparison.^24,29,37,40,42,59,67,68,70,81,82,91,98–100,110,117,118,120–144^

In contrast, many earth-abundant metal-based catalysts show large FA fractions, particularly for Ni-, Co-, Cu-, and Mn-based oxide or oxyhydroxide systems. This trend suggests that, under strongly oxidative surfaces or high anodic potentials, initially adsorbed glycerol-derived intermediates are more likely to undergo C–C cleavage and deep oxidation. Importantly, several exceptions clarify the role of adsorption-mode regulation. Ni_3_Sn, for example, shows enhanced GLCA formation, consistent with Sn weakening Ni–C interactions, preferentially destabilising multi-carbon bound intermediates and favouring predominantly mono-carbon binding. Therefore, Fig. 9 supports a refined mechanistic picture: adsorption configuration establishes the initial C3-preserving or C–C cleaving tendency, while surface oxidation state, potential, pH, and interfacial chemistry determine whether C3 intermediates are preserved or converted into C2/C1 products.

## 5. Dehydrogenation governed by surface active species

Adsorbed glycerol is subsequently activated through electrochemical dehydrogenation, giving rise to the GLD and DHA branches shown in Scheme 1. These C3 intermediates constitute an early branching point and may subsequently undergo further oxidation, chemical transformation or C–C cleavage. Section 5 examines two connected aspects of this activation step. First, reported activity trends and calculated barriers associated with glycerol dehydrogenation are discussed in relation to OH* formation energetics and surface coverage. Second, GLD/DHA branching and C3 selectivity are examined in relation to glycerol coordination with electrolyte species, surface redox chemistry and intermediate stabilisation. Together, these discussions connect catalyst composition and the interfacial environment with the initial activation of glycerol and the formation of C3 intermediates. Dehydrogenation follows the adsorption step discussed in Section 4, while the downstream transformations of GLD and DHA also depend on their stability, desorption and reaction environment.

### 5.1. Formation of surface OH* and its role in dehydrogenation kinetics

Here, OH* denotes a surface-adsorbed hydroxyl species, and the asterisk indicates adsorption on the catalyst surface. This notation does not encompass lattice oxygen, high-valent metal oxo/oxyhydroxide species or the redox-active sites of oxyhydroxides. On metals and alloys, OH* can form *via* electrochemical water activation or hydroxide adsorption, with its formation energetics influenced by the metal–oxygen interaction strength. As a result, different metals and alloys exhibit distinct OH* adsorption energies and coverage under different working potentials. These variations regulate interfacial hydroxyl availability and can influence the kinetics of glycerol dehydrogenation.

The interfacial availability of OH* can be regulated either by balancing competitive adsorption or by stabilizing hydroxyl species at oxophilic sites. On Pt-based catalysts, the incorporation of Ni modifies the electronic structure of surface Pt atoms and alters the adsorption strengths of CO* and OH* species. As shown in Fig. 10a, surface Ni atoms readily form Ni(OH)_*x*_ islands under alkaline conditions,^91^ but these islands become strongly covered by glycerol-derived adsorbates, blocking the sites for OH* adsorption. Instead, the enhancement in catalytic performance was attributed primarily to an electronic effect, whereby Ni adjusts the binding energies of key intermediates on adjacent Pt sites and alleviates surface poisoning while maintaining sufficient oxophilicity for glycerol oxidation. By contrast, the incorporation of surface Bi species markedly promotes OH* adsorption on Pt.^120^ Cyclic voltammetry shows more pronounced features associated with OH* adsorption/desorption on Bi@Pt than on bare Pt (Fig. 10b), consistent with increased surface OH* coverage. DFT calculations further demonstrated that Bi(OH)_3_-like moieties anchored on Pt stabilize surface hydroxyls and lower the rate-limiting barrier for glycerol oxidation.

**Fig. 10:**
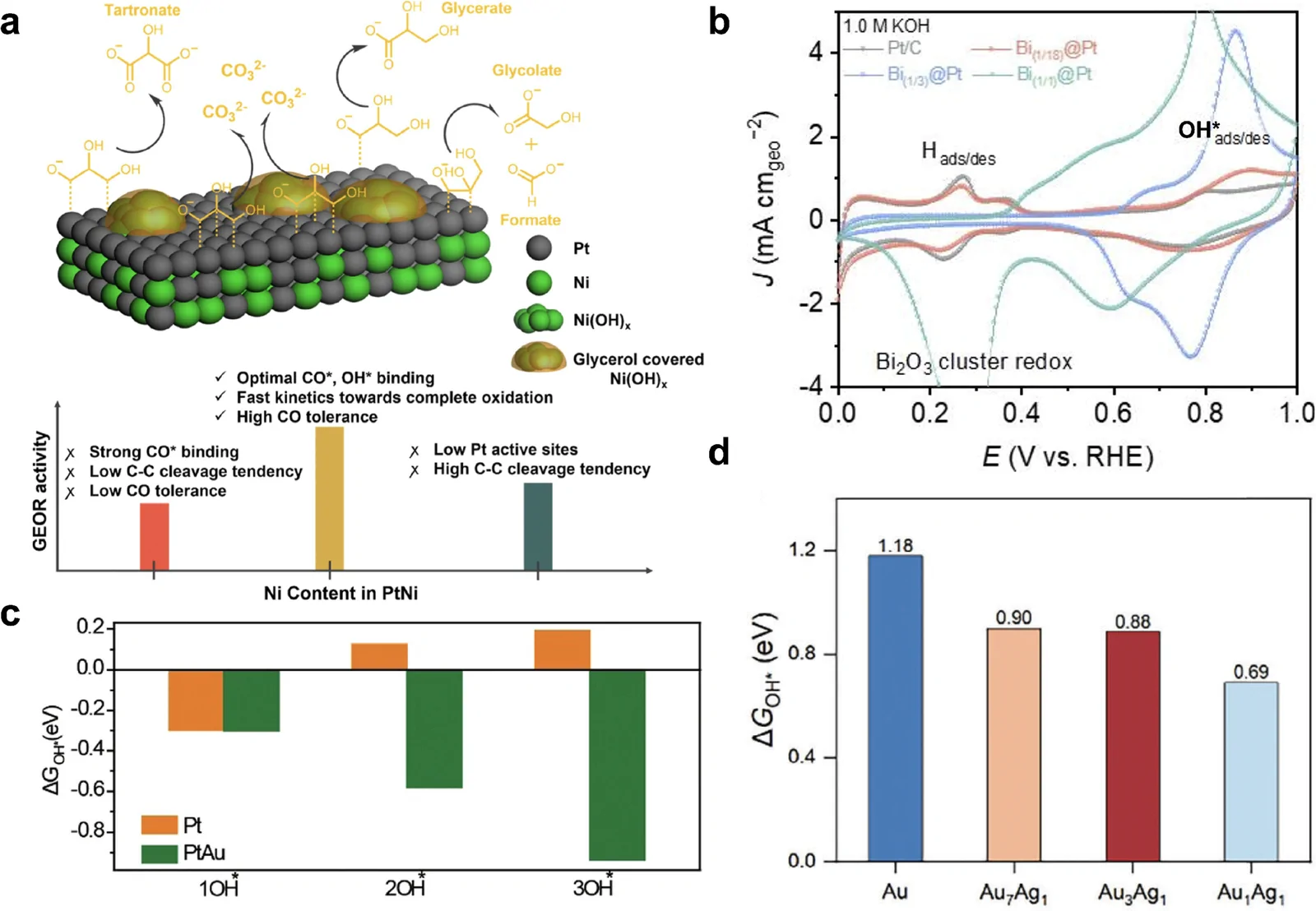
(a) Illustration of the electronic effect in PtNi electrocatalysts (green: Ni, grey: Pt). Reproduced from Luo *et al.*^91^ under the terms of the Creative Commons Attribution 4.0 International License (CC BY 4.0). Copyright 2022 The Authors, published by American Chemical Society. (b) Cyclic voltammogram of Pt-based catalysts in 1 M KOH. Reproduced with permission from Oh *et al.*^120^ Copyright 2025 The Authors, under exclusive license to Springer Nature Limited. (c) Gibbs free formation energy (Δ*G*_OH*_) on the PtAu alloy and pure Pt electrodes. Reproduced from ref. 117 with permission from the Royal Society of Chemistry. (d) Formation free energy of OH* (Δ*G*_OH*_) over the Au, Au_7_Ag_1_, Au_3_Ag_1_, and AuAg_1_ surfaces. Reproduced with permission from Yan *et al.*^42^ Copyright 2024, Wiley-VCH GmbH.

A second mode of OH* regulation involves lowering its formation energy through alloy-induced electronic modulation. In Au–Ag alloys, Ag incorporation significantly reduces the free energy barrier for OH* formation (Fig. 10d).^42^ DFT calculations further revealed that Au–Ag alloys exhibit enhanced adsorption of glycerol-derived alkoxide species compared with pure Au, and experimental measurements confirmed higher glycerol adsorption capacities and increased signals associated with reactive oxygen species. In this context, the accelerated dehydrogenation of glycerol originates from the synergistic enhancement of alkoxide adsorption and OH* availability rather than from hydroxyl generation alone. The same energetic principle applies to PtAu alloys.^117^ Introducing Au into Pt alters the electronic structure of surface Pt atoms, and markedly facilitates the formation of OH* on PtAu surfaces at higher hydroxyl coverages. This effect is evidenced by the increasingly more negative Δ*G*_OH*_ for the second and third OH* on PtAu compared with pure Pt (Fig. 10c).^117^ This enhanced hydroxyl availability enables more efficient coupling between adsorbed glycerol intermediates and OH*, thereby suppressing surface poisoning and sustaining higher dehydrogenation rates, which provides a kinetic basis for the observed preference towards C3-preserving oxidation pathways.

Taken together, these alloy systems regulate OH* at two connected levels: its interfacial availability, determined by competitive adsorption and stabilization at oxophilic sites, and its formation energetics, controlled by alloy-induced electronic modulation. Because both levels remain coupled to the adsorption of glycerol-derived species, OH* formation should not be treated as an isolated descriptor. Instead, its influence on dehydrogenation kinetics depends jointly on electronic structure, ensemble effects and competitive adsorption.

### 5.2. Control towards C3 intermediates

Beyond the OH*-assisted processes discussed above, the nature of the active surface sites also influences the initial dehydrogenation pathway and GLD/DHA branching. In glycerol electrooxidation, oxidation can proceed either through the terminal hydroxyl group to form GLD or through the secondary hydroxyl group to form DHA. This initial branching influences whether the C3 species undergo further oxidation or C–C cleavage and thereby shapes the overall product distribution. Importantly, the preference for GLD or DHA formation is not solely dictated by the intrinsic properties of the catalyst surface. Instead, it reflects the combined effects of glycerol coordination with electrolyte species, surface redox chemistry and intermediate stabilisation.

Borate complexation with glycerol and DHA can redirect the initial dehydrogenation site and stabilize the resulting C3 intermediate. In borate electrolytes, glycerol forms anionic borate-glycerol complexes, leading to enhanced catalytic activity and markedly increased C3 selectivity (Fig. 11a).^62^ Under these conditions, DHA becomes the dominant liquid product, indicating preferential oxidation at the secondary hydroxyl group. Increasing borate concentration further promotes DHA formation while suppressing the formation of more oxidized products, suggesting that coordination effects influence both the initial dehydrogenation site and the stability of C3 intermediates. This interpretation is supported by ^11^B NMR evidence for borate–DHA complex formation, which was proposed to protect DHA from further oxidation.

**Fig. 11:**
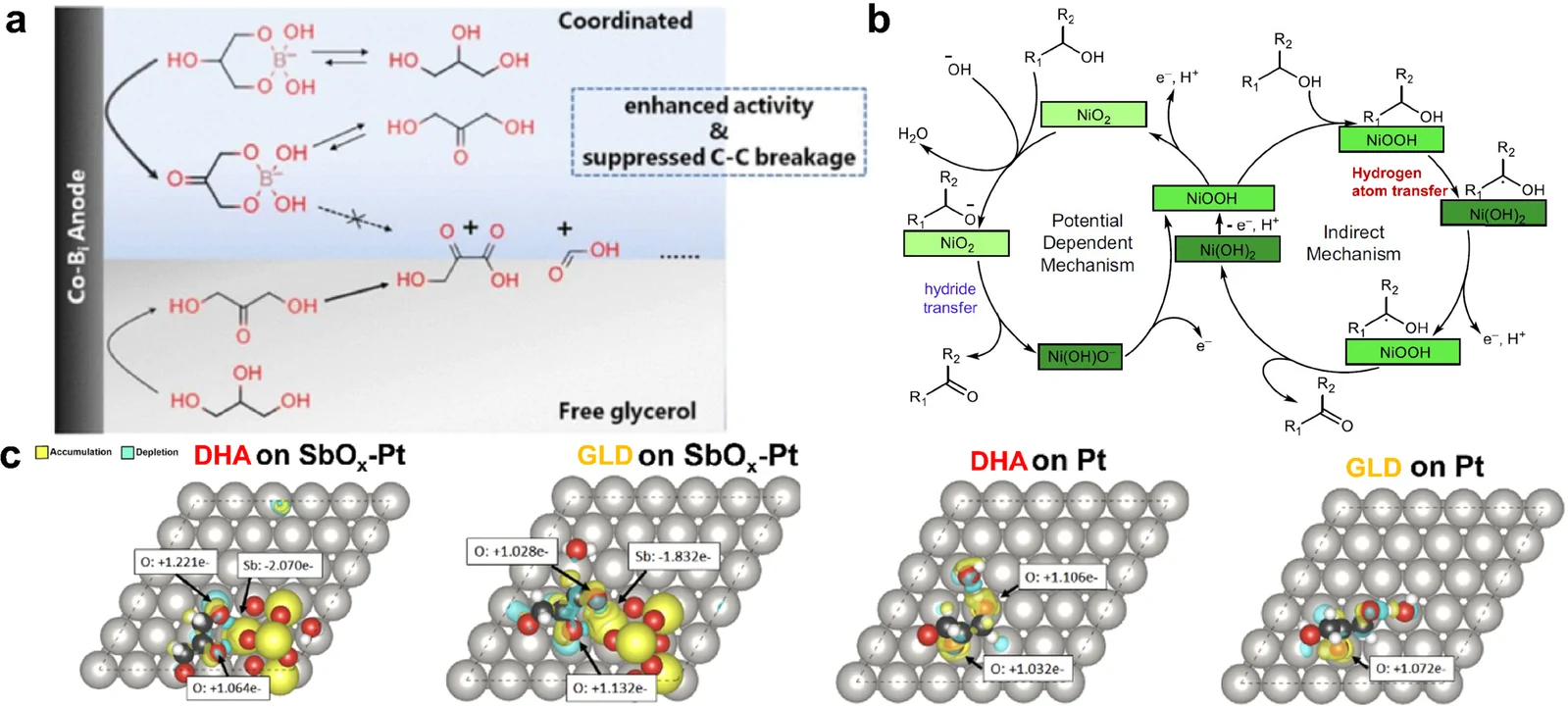
(a) Schematic illustration of the role of borate complexation in glycerol oxidation on a Co–Bi anode. Reproduced with permission from Huang *et al.*^62^ Copyright 2021, American Chemical Society. (b) Simplified representations of two possible alcohol dehydrogenation mechanisms on NiOOH electrodes. R1, R2 = aliphatic carbon chain, aromatic carbon chain, or H. For 2° alcohols, oxidation stops at the ketone product. For 1° alcohols, oxidation may proceed to the carboxylic acid *via* analogous oxidation of the hydrated geminal diol form of the initially formed aldehyde. Reproduced from Goetz *et al.*^59^ under the terms of the Creative Commons Attribution 4.0 International License (CC BY 4.0). Copyright 2022 The Authors, published by Springer Nature. (c) DHA* and GLD* on Pt-SbO_*x*_ and DHA* and GLD* on a Pt surface. Silver, red, dark grey, white, and yellow balls represent Pt, O, C, H, and Sb, respectively. Reproduced with permission from Kim *et al.*^148^ Copyright 2023 The Authors, published by Elsevier.

Beyond electrolyte effects, catalyst-dependent surface redox chemistry also governs site-selective dehydrogenation. Glycerol oxidation on NiOOH has been proposed to involve two distinct dehydrogenation pathways depending on the electrode potential and electrolyte alkalinity (Fig. 11b).^59,145^ At high alkaline concentration, deprotonation of glycerol facilitates a hydride-transfer pathway mediated by high-valence Ni^4+^ species, which preferentially oxidizes primary alcohol groups and promotes subsequent C–C break. In contrast, lowering the electrolyte pH suppresses deprotonation and shifts the reaction towards a radical-mediated hydrogen atom transfer mechanism involving Ni^3+^ centres. Because the secondary C–H bond is energetically easier to cleave than the primary one, this mechanistic switch directs the initial oxidation towards the secondary hydroxyl group, resulting in enhanced DHA formation.^146^ However, no glycerol-specific kinetic isotope effect has directly demonstrated the proposed switch between these two molecular mechanisms.^59,145,147^ These results show that pH and potential regulate the initial oxidation selectivity on NiOOH, although the precise hydrogen-transfer mechanism remains condition-dependent.

Surface-site regulation provides an additional route to tune site selectivity by stabilizing specific C3 intermediates. The introduction of amorphous SbO_*x*_ onto Pt nanoparticles markedly increases DHA selectivity relative to bare Pt, indicating preferential secondary-hydroxyl oxidation.^148^ DFT calculations revealed that SbO_*x*_ on Pt(111) makes the DHA-forming pathway thermodynamically more favourable than the GLD-forming pathway (Fig. 11c), consistent with experimental observations. This selectivity shift originates from the preferential stabilization of DHA-related intermediates at SbO_*x*_–Pt interfacial sites, where oxygen-containing Sb species selectively lower the free energy of secondary hydroxyl oxidation relative to primary hydroxyl pathways. This intermediate-selective stabilization highlights that pathway control can be achieved not only by tuning adsorption strength but also by manipulating the relative stability of competing intermediates.

These studies indicate that C3 product selectivity depends on coordination between electrolyte species and glycerol or its intermediates, alongside surface redox chemistry and intermediate stabilisation. Rather than being an intrinsic property of a single catalyst, C3 selectivity emerges from how the reaction environment and surface chemistry jointly shape the first dehydrogenation step. At the same time, modifying these factors can also shift the rate-limiting step, as stabilization of specific C3 intermediates can hinder their desorption or further transformation. This understanding provides a mechanistic basis for steering glycerol oxidation towards selective C3 products through rational control of electrolyte composition and catalyst surface structure.

## 6. Chemical steps coupled with electrooxidation

Scheme 1 places these chemical transformations downstream of GLD and DHA formation, primarily along pathway A towards LA. Section 6 examines how the chemical reactivity of these C3 carbonyl intermediates shapes the product distribution measured during electrolysis. Under alkaline conditions, isomerization, dehydration and Cannizzaro rearrangement provide a route to LA,^73,149–151^ whereas continued electrooxidation of GLD-derived intermediates favours GLCA formation.^32,54,152^ The relative GLCA/LA distribution is therefore used qualitatively to assess the balance between electrochemical oxidation and subsequent chemical conversion. The section then considers how oxide or Lewis-acidic sites and the local hydroxide environment can shift this balance towards LA formation. These chemical processes operate alongside electrooxidation: dehydrogenation supplies the C3 carbonyl intermediates, while solution- and interface-mediated reactions can redistribute them before final product analysis. Potential contributions to C–C cleavage are considered separately in Section 7.

### 6.1. Isomerization, dehydration and Cannizzaro rearrangement

First, GLD and DHA are interconverted through base-catalysed aldose-ketose isomerization mediated by enediol-type intermediates.^153^ In alkaline media, α-deprotonation facilitates the formation of enediolate species, enabling rapid interconversion between aldehyde and ketone forms. GLD may also exist as hydrated geminal-diol species, with the corresponding gem-diolate becoming relevant under alkaline conditions.^60,61^ A cyclic hemiacetal dimer has been identified in electrochemical studies of isolated glyceraldehyde, but its dominant participation during GOR has not been established.^154^ As a result, the GLD/DHA distribution measured after electrolysis may not directly reflect the initial dehydrogenation selectivity at the electrode, because the two intermediates can interconvert after entering the electrolyte. This chemical interconversion therefore complicates the assignment of the initial electrochemical pathway based solely on *ex situ* product analysis.

Secondly, DHA and GLD can undergo base-catalysed dehydration to form intermediates such as 2-hydroxypropenal, which can undergo keto-enolic tautomerization to pyruvaldehyde. This dehydration step is initiated by hydroxide-assisted α-deprotonation, leading to enolate intermediates, which subsequently undergo β-elimination to form α,β-unsaturated carbonyl species. The formation of α-dicarbonyl intermediates constitutes a crucial branching step, as these species serve as highly reactive precursors for Cannizzaro rearrangements under alkaline conditions.

Third, pyruvaldehyde can undergo Cannizzaro rearrangements under alkaline conditions whereby the hydroxide ions attack the carbonyl carbon, triggering intramolecular hydride transfer and molecular rearrangement that ultimately yield LA. Importantly, this pathway does not require direct electron transfer at the electrode surface but proceeds through non-faradaic steps once the appropriate carbonyl precursors are formed. LA selectivity should therefore not be attributed solely to an electrode-catalysed oxidation step. Instead, it reflects both the electrochemical formation of GLD/DHA and their subsequent base-catalysed conversion in the electrolyte.

These chemical pathways are reflected in the experimentally observed differences in C3 product selectivity on noble-metal catalysts. As shown in Fig. 12, literature data for Pt-, Pd-, Au-, and Ag-based catalysts exhibit distinct preferences towards GLCA and LA formation, indicating that these catalysts tend to favour different C3 oxidation routes. In general, Ag- and Pd-based systems more frequently appear in the GLCA-rich region, whereas Au- and Pt-based systems are more often associated with higher LA selectivity under alkaline conditions. This difference likely reflects how different noble-metal surfaces regulate the early oxidation of glycerol and the subsequent fate of C3 intermediates. Catalysts that favour direct electrooxidation of GLD-derived intermediates tend to give higher GLCA selectivity, whereas catalysts that more readily couple electrochemical oxidation with subsequent alkaline chemical steps can show higher LA formation.

**Fig. 12:**
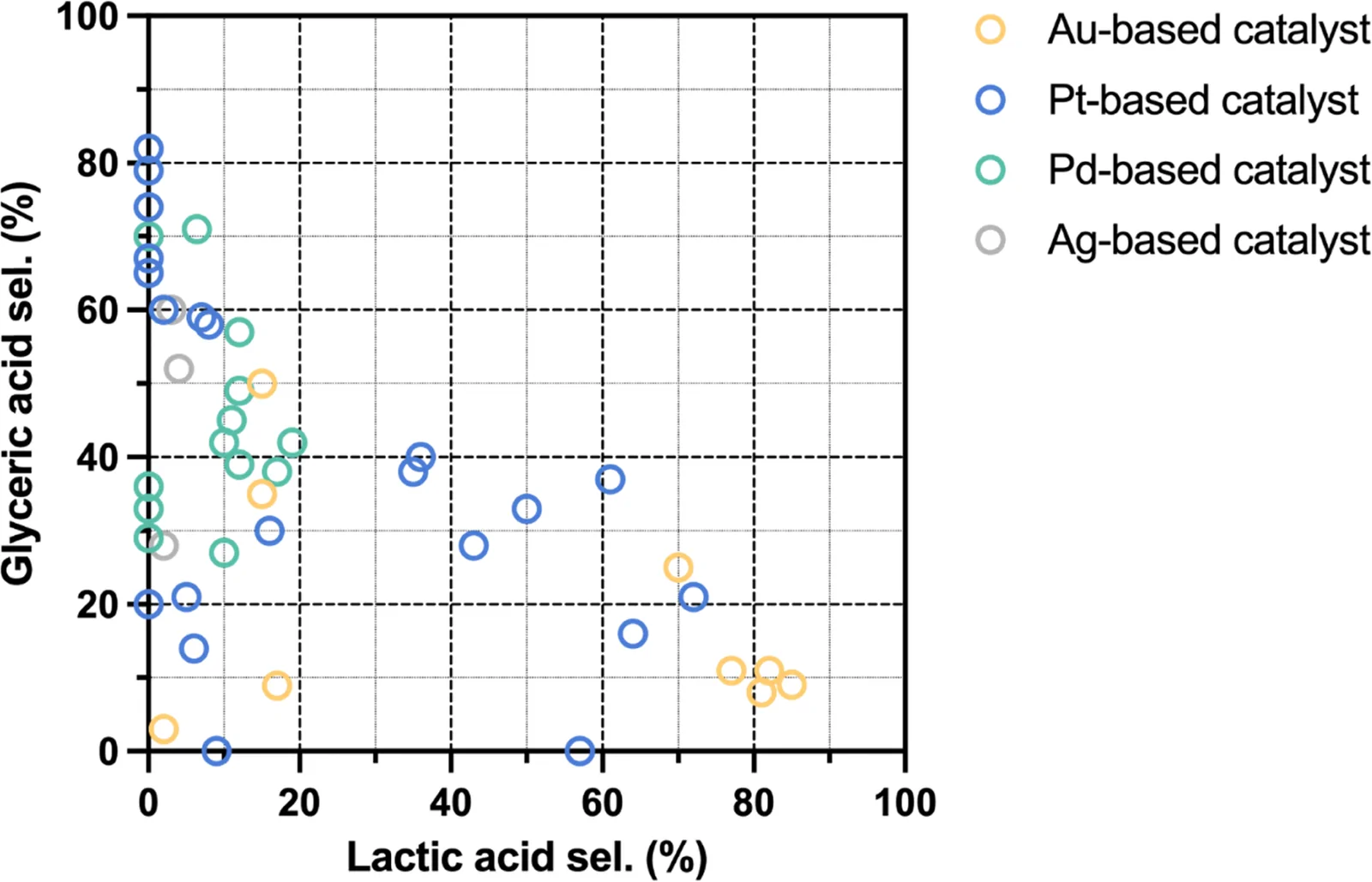
Literature distribution of GLCA and LA selectivity during glycerol electrooxidation over noble-metal-based catalysts. Each point represents one reported catalyst system compiled from the studies summarized in Fig. 9 (the detailed catalyst systems and literature sources are provided in the corresponding references summarized in Fig. 9).^24,29,37,40,42,59,67,68,70,81,82,91,98–100,110,117,118,120–144^ Because the data were obtained under different experimental conditions, including different catalyst structures, supports, electrolytes, and applied potentials, the figure is intended to illustrate qualitative trends in C3 product distribution rather than enable direct quantitative comparison.

In summary, the sequential isomerization, dehydration, and Cannizzaro rearrangement constitute a set of chemical steps that proceed alongside electrooxidation.^64^ These reactions rationalize why LA and other C3 products frequently prevail under strongly alkaline conditions, even when they are not directly formed in the initial electrochemical step. More importantly, they demonstrate that glycerol electrooxidation cannot be interpreted solely in terms of surface electrochemistry; instead, the observed selectivity emerges from the interplay between electrochemical oxidation and subsequent chemical transformations in the electrolyte.

### 6.2. The role of metal oxides and Lewis-acidic supports

Metal oxides and Lewis acid supports can strongly influence glycerol electrooxidation by intervening in the chemical transformations of C3 intermediates.^121,155–157^ Rather than acting as inert supports, the oxide domains often participate in a tandem process as co-catalysts, in which metal sites generate C3 carbonyl intermediates electrochemically, while oxide sites promote their subsequent chemical conversion.

In Pt/oxide tandem systems, Pt catalyses the electrochemical formation of GLD and DHA, whereas acidic sites on CeO_2_, TiO_2_ and γ-Al_2_O_3_ were proposed to bind DHA and promote DHA dehydration to pyruvaldehyde; the latter subsequently undergoes OH^−^-promoted Cannizzaro rearrangement to LA. Across this series of oxide supports, LA yield was found to correlate with surface acid-site density (Fig. 13a).^133^ Similar behaviour was reported in Pt/C-zeolite composites,^131^ where Lewis-acid sites were proposed to stabilize and transform DHA, strengthening the chemical pathway that Pt alone does not favour (Fig. 13b).

**Fig. 13:**
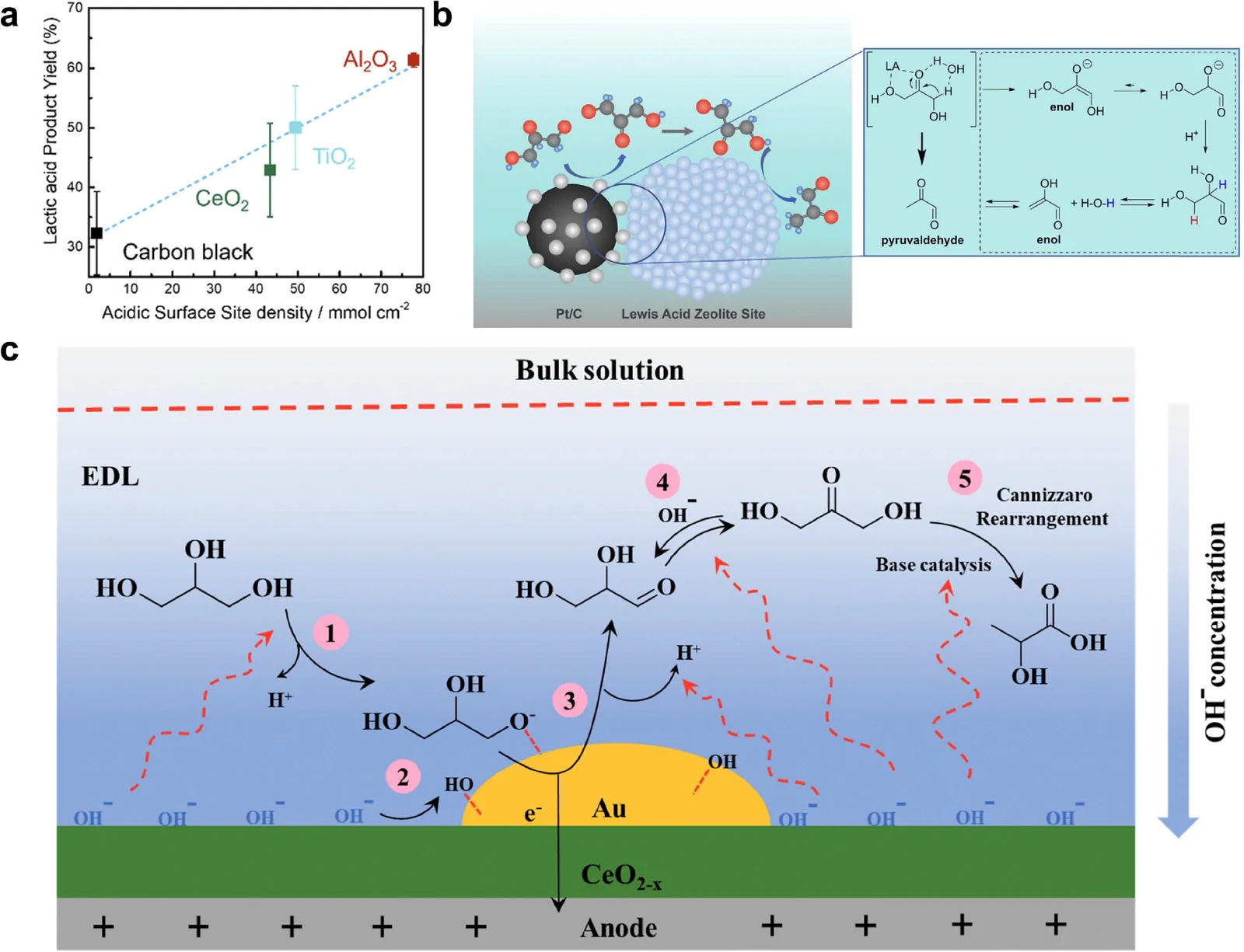
(a) The correlation between LA yield % and the acidic surface site density determined by NH_3_-TPD. Reproduced from Luo *et al.*^133^ under the terms of the Creative Commons Attribution 3.0 Unported Licence (CC BY 3.0). Copyright 2025 The Authors, published by the Royal Society of Chemistry. (b) Illustration of the GLY → DHA → pyruvaldehyde transformation at Pt/C and zeolite microenvironment interface. Reproduced from Aslam *et al.*^131^ under the terms of the Creative Commons Attribution 4.0 International License (CC BY 4.0). Copyright 2023 The Authors, published by IOP Publishing Ltd. (c) Schematic illustration of the OH^−^ enrichment effect on the Au/CeO_2−*x*_ surface for GOR. Reproduced with permission from Huang *et al.*^122^ Copyright 2024, Wiley-VCH GmbH.

Beyond promoting specific reactions, oxides can also regulate the local interfacial environment. As shown in Fig. 13c, in Au/CeO_2−*x*_ catalysts,^122^ the Lewis acid oxide support was shown to influence hydroxide transport and local pH near the anode surface. By facilitating hydroxide accumulation and mitigating local acidification, CeO_2−*x*_ enhances both activity and LA selectivity compared with bare Au. These results indicate that oxide supports can affect glycerol oxidation not only through catalytic chemistry, but also through modulation of the interfacial reaction environment.

These oxide- and zeolite-assisted systems should therefore be distinguished from a direct surface electrochemical route to LA. They are interface-assisted chemical pathways: solid acidic sites can bind C3 intermediates and promote their non-faradaic conversion near the catalyst interface, whereas in electrolyte-dominated pathways the desorbed intermediates undergo the corresponding transformations in the alkaline electrolyte. Thus, the two cases differ primarily in the location and catalytic control of the chemical step, rather than in whether the final LA-forming conversion involves electron transfer. To the best of our knowledge, a predominantly direct faradaic surface pathway to LA has not yet been demonstrated.

Overall, metal oxides and Lewis acid supports provide an additional layer of control over glycerol electrooxidation by coupling electrochemical and chemical processes. Their influence on both intermediate transformation and interfacial conditions offers a mechanistic basis for understanding why oxide-modified catalysts often exhibit enhanced selectivity towards C3 products.

## 7. C–C bond cleavage: pathway towards C2 and C1 products

Pathway C in Scheme 1 represents C–C bond cleavage and marks the transition from C3-preserving oxidation to the formation of C2 and C1 products. Section 7 examines how applied potential and electrolyte pH shift the balance between these product pathways. The extent of C–C cleavage is inferred from decreasing C3 selectivity and the corresponding formation of C2/C1 products, while faradaic efficiency and carbon balance provide additional evidence. The potential dependence is interpreted through changes in surface oxidation state and hydroxyl or oxygen coverage, whereas the pH dependence reflects changes in catalyst surface chemistry and the stability of intermediates. These trends connect C–C cleavage with the adsorption and surface-oxygen chemistry discussed in Sections 4 and 5. At high anodic potentials, surface oxide formation can be associated with both deeper oxidation and activity loss, providing a link to catalyst deactivation in Section 8.

### 7.1. The effect of oxidation potential on C–C bond cleavage

Increasing anodic potential can make consecutive oxidation and C–C cleavage more competitive by changing the catalyst surface state, hydroxyl/oxygen coverage and the reactivity of adsorbed intermediates.^158–160^ This potential-dependent shift has been widely observed across both noble-metal and earth-abundant catalytic systems and is therefore regarded as a general trend in GOR, although its magnitude and onset depend strongly on the catalyst surface state and reaction environment.^152^ The adsorption preferences discussed in Section 4 therefore provide an initial pathway bias, while the potential-dependent surface chemistry determines how readily the resulting intermediates undergo further conversion.

Consecutive oxidation of C3 intermediates provides one route to increased C2/C1 formation. On Pt-based catalysts, relatively low potentials can favour C3 products, whereas increasing potential promotes more extensive oxidation associated with changes in surface hydroxyl and oxygen species (Fig. 14a).^98,124,161,162^ Theoretical studies further indicate that potential-dependent changes in oxygen coverage on Pt surfaces can modify the adsorption strength and relative energetics of glycerol-derived intermediates, thereby changing the preferred reaction pathway and the step that requires the highest potential.^85^ Related behaviour has been reported on Au, where GLCA formed through the GLD branch undergoes further oxidation to GCA and FA as the potential increases.^161^ These examples connect potential-dependent selectivity to the subsequent consumption of C3 intermediates, rather than solely to changes in the initial glycerol activation step.

**Fig. 14:**
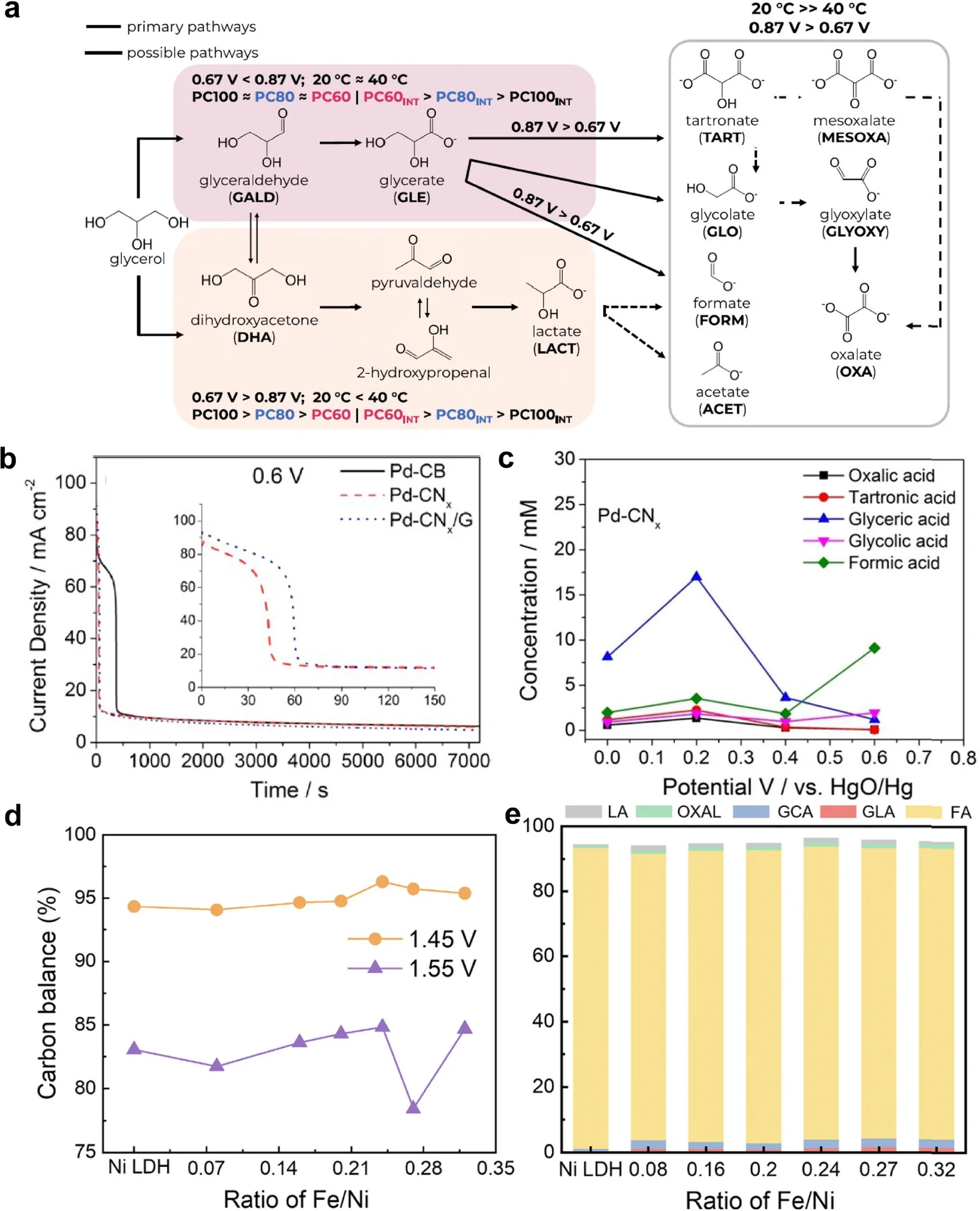
(a) GOR mechanism on Pt_*x*_Co_100−*x*_ NPs in an alkaline medium. Reproduced from Terekhina *et al.*^124^ under the terms of the Creative Commons Attribution 4.0 International License (CC BY 4.0). Copyright 2024 The Authors, published by American Chemical Society. (b) Chronoamperometry curves of Pd catalysts at 0.6 V. Reproduced with permission from Wang *et al.*^164^ Copyright 2015, American Chemical Society. (c) The concentration of products formed over Pd catalysts produced at different applied potentials. Reproduced with permission from Wang *et al.*^164^ Copyright 2015, American Chemical Society. (d) Carbon balance at applied potentials of 1.45 and 1.55 V as a function of Fe doping ratio in Ni LDH. Reproduced with permission from Kim *et al.*^165^ Copyright 2024, American Chemical Society. (e) Selectivity of products at 1.45 V *vs.* RHE as a function of Fe doping ratio in Ni LDH. Reproduced with permission from Kim *et al.*^165^ Copyright 2024, American Chemical Society.

Surface oxidation can also change selectivity while reducing overall activity. In a Pd-based system, anodic conditions associated with Pd oxide formation produced a pronounced current decrease, while FA became the dominant product with a reported selectivity of approximately 70% (Fig. 14b and c).^163,164^ The observation links surface-state evolution with a shift towards C–C cleavage, but also shows that a larger fraction of deeply oxidised products does not necessarily imply higher overall catalytic activity. The consequences of these surface changes for sustained operation and regeneration are discussed in Section 8.

At higher potentials, carbon balance and competing OER become important to pathway interpretation. In Ni-based LDH systems, operation at 1.55 V *versus* RHE was accompanied by lower carbon balance than at 1.45 V under the reported conditions (Fig. 14d).^165^ Formation of undetected CO_2_ or carbonate was suggested as a possible explanation, although the missing carbon does not itself identify the products responsible. Fe incorporation also shifted the potential required for FA formation and changed the product distribution (Fig. 14e). Because OER can consume current and compete for surface sites in this operating regime, total current must be considered alongside product-specific faradaic efficiencies and carbon balances.

The influence of applied potential on product selectivity is further illustrated in Fig. 15. By co-plotting C3 selectivity data with representative glycerol-containing LSVs, the relationship between product selectivity and electrochemical behaviour under reaction-relevant conditions becomes more evident. A clear distinction can be observed between noble-metal and earth-abundant metal catalytic systems. Noble-metal catalysts predominantly operate in lower potential regimes, where the anodic current increases more gradually, and relatively high C3 selectivity is maintained over a broad potential window. In contrast, earth-abundant metal catalysts are more frequently evaluated at higher anodic potentials, which are accompanied by a rapid rise in anodic current, where C3 selectivity is markedly reduced, with many data points clustered at low C3 fractions. Importantly, the potential range over which C3 selectivity begins to decline broadly coincides with the onset of significant anodic current in the corresponding LSVs, indicating a close association between changes in the electrochemical response and selectivity loss. Because the compiled data originate from different catalyst structures, electrolytes, loadings, reaction times, and cell configurations, Fig. 15 should not be interpreted as demonstrating a universal monotonic dependence of C3 selectivity on potential. Rather, together with potential-resolved studies on individual catalyst systems, it illustrates the broader tendency for C–C cleavage pathways to become increasingly competitive at higher anodic potentials.

**Fig. 15:**
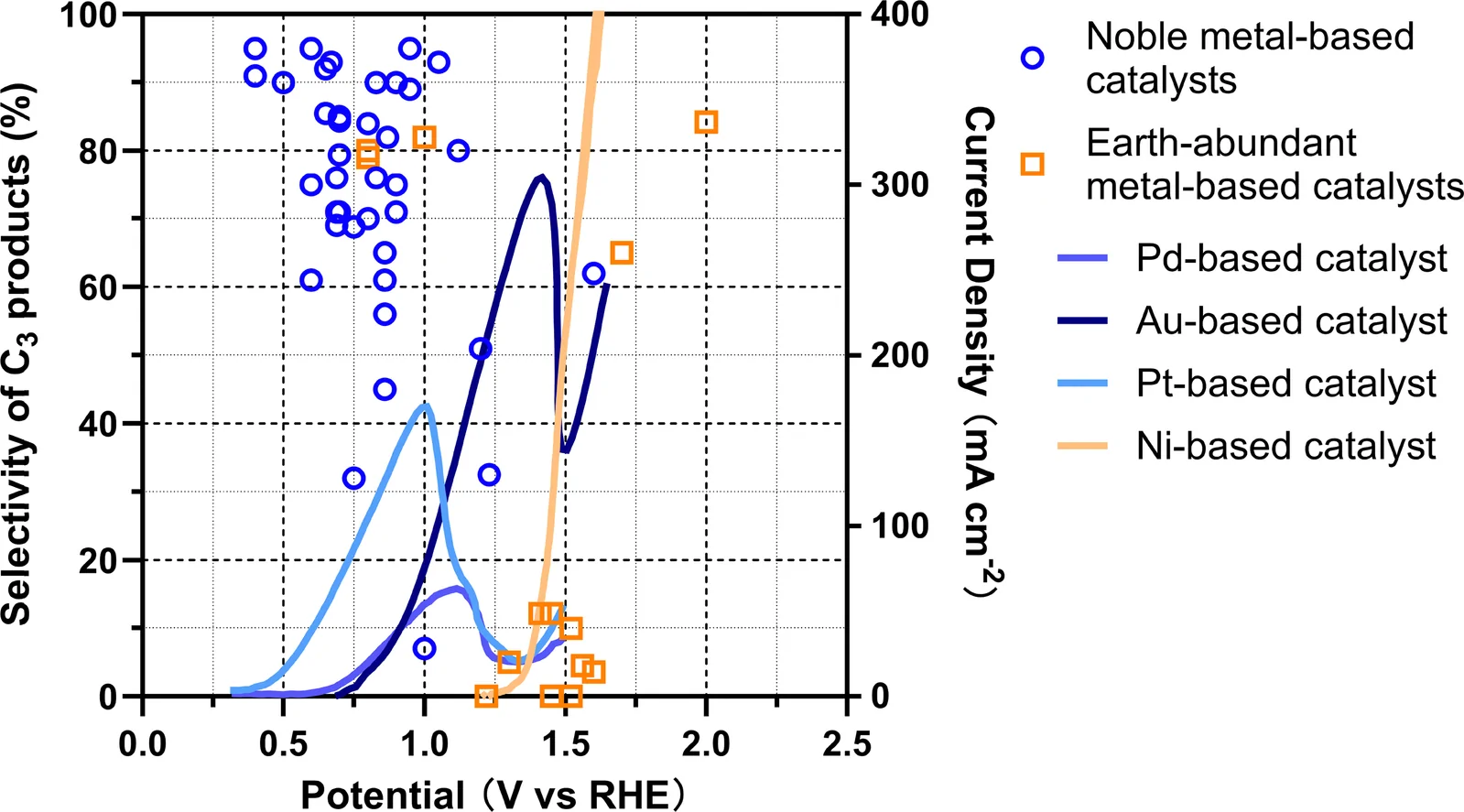
Dependence of C3 product selectivity on applied potential during glycerol electrooxidation over noble-metal- and earth-abundant-metal-based catalysts. Scatter points were compiled from literature reports summarized in Fig. 9,^24,29,37,40,42,59,67,68,70,81,82,91,98–100,110,117,118,120–144^ each point representing one reported catalyst system under its corresponding testing conditions. Representative glycerol-containing LSVs were replotted with permission from the literature, including Pd-based catalysts from Xia *et al.*^134^ (0.5 M KOH, 0.3 M GLY, Pd catalyst) Copyright 2024, Wiley-VCH GmbH, Au-based catalysts from Yan *et al.*^113^ (3 M KOH, 0.3 M GLY, Au catalyst) Copyright 2024, American Institute of Chemical Engineers, Pt-based catalysts from Huang *et al.*^122^ (1 M KOH, 0.5 M GLY, Pt catalyst) Copyright 2024, Wiley-VCH GmbH, and Ni-based catalysts from Kim *et al.*^165^ (1 M KOH, 0.1 M GLY, NiFe catalysts) Copyright 2024, American Chemical Society. Because the data were collected with different catalyst structures, loadings, supports, electrolytes, and cell configurations, the plot is intended to illustrate qualitative trends rather than enable direct quantitative comparison.

### 7.2. pH effect on C–C cleavage

Although alkaline electrolytes provide the principal evidence base for this review, comparisons across acidic, neutral and alkaline media show that pH is a mechanistic variable that can redirect the GOR pathway. Changes in pH alter glycerol deprotonation, hydroxyl availability, catalyst oxidation state and the stability of intermediates in the electrolyte. These effects influence both the initial oxidation pathway and the subsequent balance between C3 retention and C–C cleavage. The resulting pH dependence is catalyst-specific rather than universally monotonic. Moderate-pH conditions can suppress extensive oxidation and favour C3 intermediates in several systems, whereas strongly acidic or alkaline media may promote deeper oxidation when the corresponding oxidized catalyst surface or chemical reactions in the electrolyte promote further oxidation.

A comparative study of glycerol oxidation on Au and Pt under alkaline, neutral, and acidic conditions provides a clear experimental illustration.^162^ In alkaline media, glycerol oxidation proceeds beyond C3 oxygenates: GLCA is further oxidized to GCA and FA, indicating enhanced C–C bond cleavage under strongly basic conditions. In contrast, lowering the pH suppresses deeper oxidation and shifts the reaction towards early-stage products; under neutral conditions, GLD becomes the dominant product, particularly on Au. Under acidic conditions, Au becomes inactive, while Pt remains active but with low conversion. At sufficiently positive potentials associated with Pt surface oxidation, further conversion of GLD towards FA and CO_2_ can occur. This indicates that acidic conditions do not universally suppress C–C cleavage, as extensive oxidation can still occur on specific oxidised catalyst surfaces.

A non-monotonic dependence is particularly evident for MnO_*x*_. FA was the major product under strongly acidic conditions, at pH 1 and 4, and strongly alkaline conditions, at pH 13 and 14. At pH 7 and 10, FA faradaic efficiency decreased and C3 products became more prominent (Fig. 16a).^68^ On NiOOH, lowering pH from strongly alkaline conditions to pH 9 shifted the dominant product from FA towards DHA (Fig. 16b).^59^ Mechanistically, they attributed this pH-dependent behaviour to a shift in reaction pathways: at high pH, glycerol oxidation on NiOOH proceeds predominantly through a potential dependent mechanism, whereas at lower pH the reaction is mainly governed by a hydrogen atom transfer pathway (the pathway we mentioned in Section 5.2, Fig. 11b) (Fig. 16c and d).

**Fig. 16:**
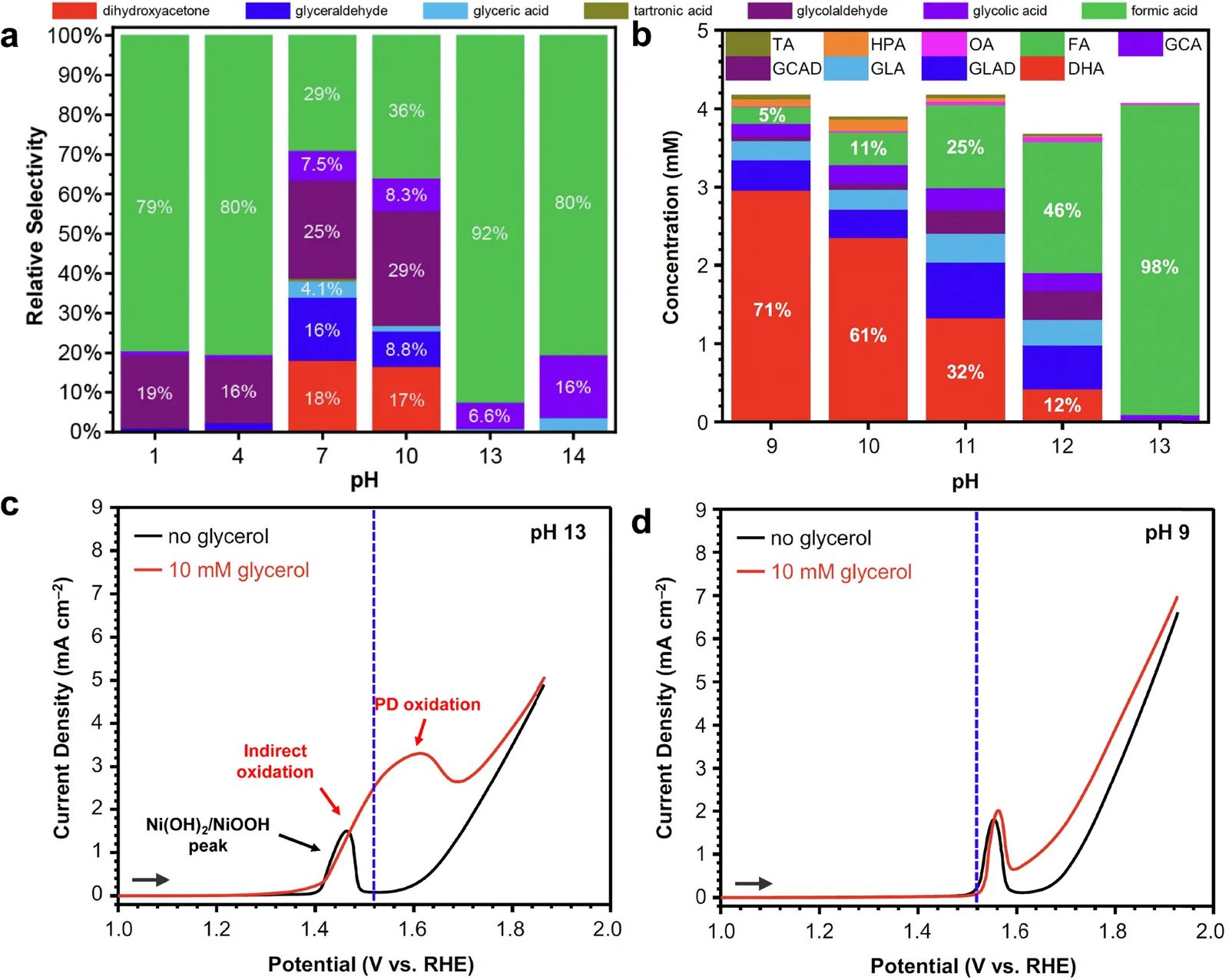
(a) The relative selectivity of glycerol oxidation results on MnO_*x*_ under various pH and 1.6 V *vs.* RHE. Reproduced with permission from Goetz *et al.*^68^ Copyright 2023, American Chemical Society. (b) Product distributions of glycerol oxidation results after electrolysis at a constant potential of 1.52 V *vs.* RHE under various pH conditions. Linear sweep voltammetry of Ni in the absence (black) and presence of 10 mM glycerol (red) obtained in an electrolyte at pH = 13 (c) and 9 (d). Reproduced from Goetz *et al.*^59^ under the terms of the Creative Commons Attribution 4.0 International License (CC BY 4.0). Copyright 2022 The Authors, published by Springer Nature.

Taken together, these comparisons show that pH does not impose a single catalyst-independent selectivity rule. Instead, it changes the dominant pathway by jointly regulating catalyst surface states, reactant deprotonation and the stability of intermediates in the electrolyte. Neutral or mildly alkaline conditions often favour the accumulation of early C3 products because surface oxidation and chemical degradation of intermediates in the electrolyte are less extensive. Strongly alkaline conditions can accelerate deprotonation and activate high-valent oxyhydroxide species, thereby promoting consecutive oxidation and C–C cleavage. Acidic media suppress the activity of catalysts such as Au, but oxidized Pt or Mn-based surfaces may still support deeper oxidation at sufficiently positive potentials.

Importantly, they also found that at pH = 13, DHA, GLD and glycolaldehyde were chemically unstable (Fig. 17). These intermediates decomposed to FA even without a catalyst. These observations show that solution-phase reactions can alter product distributions, but do not identify retro-aldol cleavage as the underlying mechanism. Neither intermediate disappearance nor FA formation alone provides specific evidence for this pathway.^152^

**Fig. 17:**
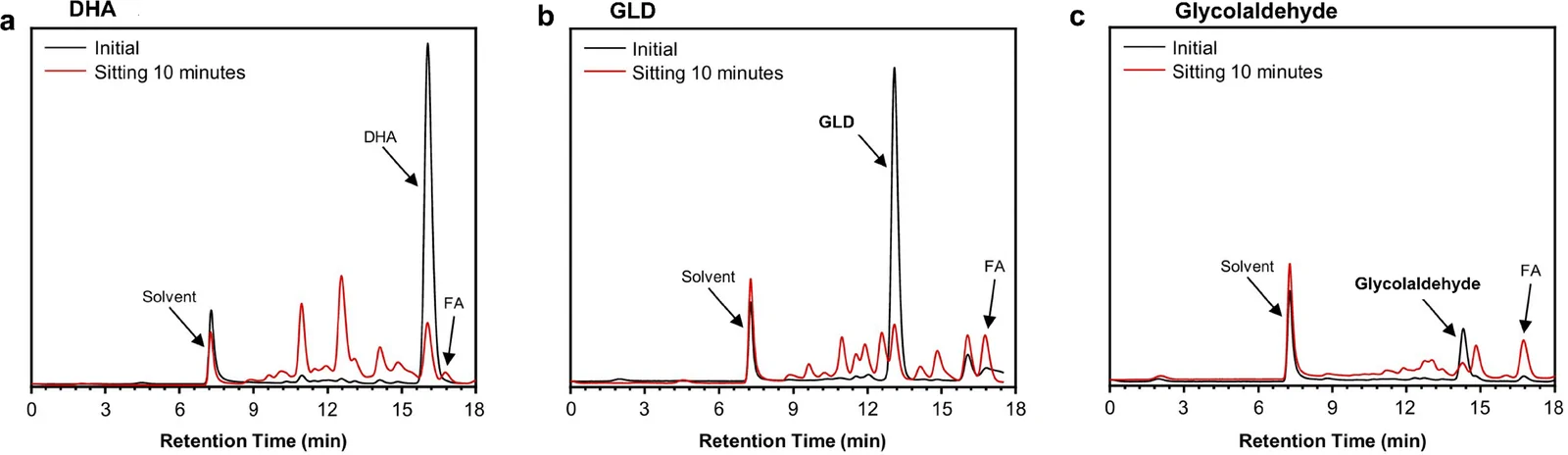
HPLC chromatograms at 205 nm showing the rapid decomposition of (a) DHA, (b) GLD, and (c) glycolaldehyde in 0.1 M KOH (pH 13) solution. Reproduced from Goetz *et al.*^59^ under the terms of the Creative Commons Attribution 4.0 International License (CC BY 4.0). Copyright 2022 The Authors, published by Springer Nature.

Evidence limiting the proposed contribution includes the observation that retro-aldolization of GLD was not observed under anaerobic conditions.^162^ This finding argues against a substantial contribution under the tested conditions, but cannot establish that retro-aldol chemistry is negligible across GOR systems. Hydrothermal studies have investigated GLD retro-aldol conversion at 350–450 °C and 25 MPa, where a radical mechanism was proposed.^166,167^ These results cannot be directly extrapolated to the milder aqueous conditions typically used for GOR. Therefore, the contribution of retro-aldol pathways to C–C bond cleavage in glycerol electrooxidation remains unresolved, and the available evidence does not establish this pathway as a general or dominant source of C1/C2 products. Quantifying its contribution requires time-resolved product analysis and carbon balances, together with catalyst-free controls under matched pH, temperature and oxygen conditions.

The discussion in Sections 4–7 shows that the balance between C3 retention and C–C cleavage depends jointly on catalyst properties, electrolyte composition and operating conditions. Table 2 brings these factors together by comparing reported C3 and C2/C1 product distributions under the corresponding experimental conditions. Selectivity and faradaic efficiency are distinguished because they describe different aspects of performance, and the differences in testing conditions should be considered when comparing catalyst systems.

**Table 2:** Cross-system comparison of catalyst, electrolyte environment, and operating conditions governing C3 retention and C–C bond cleavage during glycerol electrooxidation

Catalyst	Electrolyte	C3 and C2/C1 product distribution	Operating conditions	Ref.
A C3 retention				
hp-PtAu/NF	OH^−^ 1 M; GLY 0.5 M	(a) C3: LA 70%; GLCA 25%; (b) C3: LA 15%; GLCA 35%; TA 12%; C2/C1: —	(a) *E* = 0.4 V; rt; (b) *E* = 1.6 V; rt	117
Bi(1/3)@Pt	OH^−^ 1 M; GLY 0.5 M	C3: GLCA 74%; TA 16%; C2/C1: —	*E* = 0.5 V; rt	120
Ag(0.8)/Pt	OH^−^ 1 M; GLY 0.5 M	C3: LA 16% FE; GLCA 30% FE; TA 30% FE; C2/C1: GCA 4% FE; FA 6% FE	*E* = 0.83 V; 30 °C	118
(a) AgPt; (b) AgAu; (c) AgPd	OH^−^ 1 M; GLY 0.1 M	(a) C3: LA 3% FE; GLCA 60% FE; TA 12% FE C2/C1: GCA 6% FE; (b) C3: LA 2% FE; GLCA 28% FE; TA 21% FE C2/C1: GCA 26% FE; (c) C3: LA 4% FE; GLCA 52% FE; TA 15% FE C2/C1: GCA 12% FE	(a) *E* = 0.9 V; rt; (b) *E* = 1.2 V; rt; (c) *E* = 0.9 V; rt	37
Ni_3_Sn	OH^−^ 0.5 M; GLY 0.5 M	C3: LA 3%; GLCA 62%; C2/C1: GCA 6%; FA 26%	*E* = 1.7 V; rt	24
MnSb_2_O_6_	OH^−^ 1 M; GLY 0.01 M	C3: GLCA 82%; TA 2.2%; C2/C1: GCA 8.8%; FA 7.2%	*E* = 2.0 V; rt	68
Au_3_Ag_1_	OH^−^ 3 M; GLY 0.3 M	C3: LA 85%; GLCA 9%; TA 1%; C2/C1: GCA 2%; FA 2%	*E* = 0.95 V; rt	42
Au/Ni(OH)_2_	OH^−^ 3 M; GLY 0.3 M	C3: LA 77%; GLCA 11%; TA 1%; C2/C1: FA 4%	*E* = 0.95 V; rt	81
Au/CeO_2−*x*_	OH^−^ 1 M; GLY 0.5 M	C3: LA 81%; GLCA 8%; C2/C1: GCA 4%; FA8%	*E* = 0.83 V; rt	122
Au-CeO_2_/CNT	OH^−^ 1 M; GLY 0.5 M	C3: LA 15%; GLCA 50.0%; TA 15%; C2/C1: GCA 11%; FA 7%	*E* = 1.12 V; 60 °C	121
MoO_*x*_/Pt	OH^−^ 1 M; GLY 0.1 M	(a) C3: GLCA 79%; TA 3% C2/C1: FA 9%; (b) C3: GLCA 56%; TA 23% C2/C1: GCA 3.2%; FA 5%; (c) C3: GLCA 73%; TA 7% C2/C1: GCA 8%; FA 5%	(a) *E* = 1.0 V, rt; (b) *E* = 0.8 V, rt, 60 h; (c) *E* = 0.8 V, rt	82
Pt/C-zeolite	OH^−^ 1 M; GLY 1 M	C3: LA 57.3%; TA 2% C2/C1: GCA 36%	*j* = 20 mA cm^−2^; 60 °C	131
(a) Pt/C-Al_2_O_3_; (b) Pt/C-CeO_2_; (c) Pt/C-TiO_2_	OH^−^ 1 M; GLY 1 M	(a) C3: LA 61%; GLCA 37%; TA 2%; (b) C3: LA 43%; GLCA 28%; TA 3%; (c) C3: LA 50%; GLCA 33%; TA 2%; C2/C1: —	(a) *j* = 20 mA cm^−2^; 60 °C; (b) *j* = 20 mA cm^−2^; 60 °C; (c) *j* = 20 mA cm^−2^; 60 °C	133
Pt/FeNC	OH^−^ 8 M; GLY 5 M	C3: LA 5%; GLCA 21%; TA 64%; C2/C1: GCA 4%; FA 2%	*E* = 0.9 V; 80 °C	132
PtNi_2_	OH^−^ 1 M; GLY 0.1 M	C3: GLCA 20% FE; TA 51% FE; C2/C1: —	*E* = 0.7 V; rt	91
PdCuL/C^v^	OH^−^ 2 M; GLY 2 M	C3: GLCA 33%; TA 58%; C2/C1: FA 9%	*E* = 0.4 V; 60 °C	135
Pd/NF	OH^−^ 1 M; GLY 0.1 M	(a) C3: LA 6.4%; GLCA 70.5%; TA 8.6% C2/C1: FA 8%; (b) C3: LA 12%; GLCA 56.8%; TA 10.6% C2/C1: GCA 4%; FA 9.2%; (c) C3: LA 11%; GLCA 45%; TA 12.8% C2/C1: GCA 10%; FA 11%	(a) *E* = 0.65 V; rt; (b) *E* = 0.7 V; rt; (c) *E* = 0.75 V; rt	136
Pt@G	OH^−^ 1 M; GLY 0.02 M	(a) C3: GLCA 81.8%; TA 4.2% C2/C1: GCA 11%; FA 1%; (b) C3: LA 35%; GLCA 37.8%; TA 11.6% C2/C1: GCA 5%; FA 2.5%	(a) Pulse: 0.3 V, 0.5 s/0.7 V, 0.05 s; 10 C; rt; (b) *E* = 0.7 V; rt	127
Pt(LaO_*x*_)/C	OH^−^ 0.1 M; GLY 0.1 M	C3: GLCA 67%; TA 4%; C2/C1: GCA 5%; FA 4%	Cell voltage 0.7 V; rt	125
Pt/C	OH^−^ 0.1 M; GLY 0.1 M	C3: GLCA 64.5%; TA 2.8%; C2/C1: —	*E* not specified; rt	126
Pt_75_Fe_25_/C Polyol	OH^−^ 0.1 M; GLY 0.5 M	C3: GLCA 79%; TA 6%; C2/C1: —	*E* = 0.7 V; rt	129
Pt/NiF-3	OH^−^ 3 M; GLY 1 M	C3: LA 64%; GLCA 16%; TA 12%; C2/C1: GCA 5%; FA 1%	*E* = 0.65 V; rt	98
Pt100	OH^−^ 2 M; GLY 1 M	(a) C3: LA 72%; GLCA 21%; (b) C3: LA 36%; GLCA 40%; TA 6%; C2/C1: GCA 6%	(a) *E* = 0.67 V; 40 °C; (b) *E* = 0.87 V; 20 °C	124
(a) Pt (linear); (b) Pt (cubic); (c) Pt (hierarchical)	OH^−^ 1 M; GLY 0.1 M	(a) C3: LA 2%; GLCA 60%; TA 7% C2/C1: FA 17%; (b) C3: LA 8%; GLCA 58%; TA 5% C2/C1: FA 14%; (c) C3: LA 7%; GLCA 59%; TA 10% C2/C1: FA 18%	(a) *E* = 0.69 V; 60 °C; (b) *E* = 0.69 V; 60 °C; (c) *E* = 0.69 V; 60 °C	110
Pd-NC	OH^−^ 1 M; GLY 0.1 M	C3: LA 19%; GLCA 42%; C2/C1: GCA 20%; FA 6.4%	*E* = 0.6 V; rt	138
(a) Pd_RD_ NPs; (b) Pd_RD_ NPs; (c) Pd_CUBE_ NPs; (d) Pd_CUBE_ NPs	OH^−^ 1 M; GLY 0.1 M	(a) C3: LA 12%; GLCA 49%; TA 4% C2/C1: GCA 15%; FA 13%; (b) C3: LA 10%; GLCA 27%; TA 8% C2/C1: GCA 20%; FA 26%; (c) C3: LA 10%; GLCA 42%; TA 4% C2/C1: GCA 24%; FA 15%; (d) C3: LA 12%; GLCA 39%; TA 10% C2/C1: GCA 16%; FA 13%	(a) *E* = 0.86 V; 20 °C; (b) *E* = 0.86 V; 60 °C; (c) *E* = 0.86 V; 20 °C; (d) *E* = 0.86 V; 60 °C	139
(B) C2/C1 formation				
Ni(OH)_2_	(a) pH = 13; GLY 0.025 M; (b) pH = 12; GLY 0.025 M	(a) C2/C1: GCA 2%; FA 98%; (b) C3: GLCA 9%; TA 1% C2/C1: GCA 7%; FA 46%	(a) *E* = 1.52 V; rt; (b) *E* = 1.52 V; rt	59
Ni-NaOHP	OH^−^ 1 M; GLY 0.1 M	C3: —; C2/C1: FA 86.3% FE	*E* = 1.41 V; rt	99
MnO_*x*_	OH^−^ 1 M; GLY 0.01 M	C3: GLCA 3.6%; C2/C1: FA 81%; GCA 16%	*E* = 1.6 V; rt	68
Au_1_Cu_1_(111)	OH^−^ 1 M; GLY 0.1 M	C3: LA 17.1%; GLCA 8.9%; TA 9.5%; C2/C1: GCA 45%; FA 19.1%	*E* = 1.23 V; 40 °C	40
RA-Au/NF	1 M KOH; GLY 0.1 M	C3: LA 2%; GLCA 3%; TA 2%; C2/C1: GCA 47%; FA 47%	*E* = 1.0 V; rt	123
Platinized titanium (flow cell)	OH^−^ 1 M; GLY 2 M	C3: LA 9% FE C2/C1: GCA 36%; FE; FA 32% FE	*j* = 50 mA cm^−2^; rt	128
CNBO	OH^−^ 1 M; GLY 0.1 M	C3: GLCA 8.57% FE; C2/C1: GCA 10.3% FE; FA 79.4% FE	*j* = 50 mA cm^−2^; rt	140
CuO-Co_3_O_4_/NF	OH^−^ 1 M; GLY 0.1 M	C3: GLCA 5%; C2/C1: FA 90.3%	*E* = 1.3 V; rt	141
Cu-CuS/BM	OH^−^ 0.1 M; GLY 0.1 M	C3: —; C2/C1: FA 86.0%	*E* = 1.45 V; rt	142
CoNA-NiOH/NF-2	OH^−^ 1 M; GLY 0.1 M	C3: —; C2/C1: FA 90% FE	*E* = 1.22 V; rt	29
Ni/Co(1:2-TT)	OH^−^ 1 M; GLY 0.1 M	C3: —; C2/C1: GCA 4.8% FE; FA 80.6% FE	*j* = 10 mA cm^−2^; rt	144
NiSex/Ni NRAs	OH^−^ 1 M; GLY 0.1 M	C3: GLCA 12.2% FE C2/C1: GCA 9.24% FE; FA 71.5% FE	*E* = 1.45 V; rt	70
Ni_12_P_5_	OH^−^ 1 M; GLY 1 M	C3: —; C2/C1: FA 98% (include oxalate); individual FA fraction not resolved here	*j* = 50 mA cm^−2^; rt	100

## 8. Desorption and catalyst deactivation

Unlike pathways A–C in Scheme 1, desorption and catalyst deactivation do not constitute a separate product-forming branch but influence sustained catalytic performance across the reaction network. Section 8 focuses on two sources of performance loss: active site blocking by strongly adsorbed intermediates or poisoning species, and potential-induced surface oxidation, both of which reduce the availability and reactivity of active sites. From this perspective, deactivation reflects the dynamic balance between adsorbate accumulation, surface oxidation and active-site regeneration: desorption contributes to site renewal by removing surface-bound species, whereas electrochemical regulation determines whether poisoned or oxidized surface states persist. This discussion connects deactivation with the adsorption strength and adsorbate accumulation described in Section 4, the availability of reactive hydroxyl species discussed in Section 5, and the high-potential surface states associated with deeper oxidation in Section 7.

### 8.1. Surface poisoning mechanisms: CO and oxide formation

Catalyst deactivation is a critical issue in GOR, as it directly affects catalytic activity, product selectivity, and long-term stability. Under anodic polarization, deactivation generally arises from the coupled effects of surface poisoning and surface oxidation, which jointly reduce the availability and reactivity of active sites.

CO species can adsorb strongly on Pt surfaces and outcompete glycerol, thereby blocking sites required for glycerol and water adsorption. This site blocking partially inhibits glycerol oxidation and contributes to catalyst deactivation.^104^ In contrast to its poisoning role on Pt and other transition metals, adsorbed CO exhibits markedly different behaviour on Au. On Au(111) in alkaline media, CO can promote rather than inhibit alcohol oxidation.^168^ Specifically, CO adsorption on Au surfaces enhances the binding of hydroxyl species on neighbouring sites, thereby facilitating the oxidative dehydrogenation steps of alcohol molecules. This cooperative interaction between CO* and OH* lowers the onset potential and increases the oxidation rate of alcohols, highlighting a counterintuitive promotional effect of CO on Au catalysts. Such behaviour is fundamentally different from that on Pt, where CO adsorption generally leads to site blocking and catalytic inhibition.

Beyond poisoning by adsorbed intermediates, potential-induced surface oxidation further exacerbates catalyst deactivation. On Pt- and Pd-based catalysts, activity decay under anodic polarization is closely associated with potential-induced surface oxidation and the accumulation of strongly adsorbed intermediates, as we mentioned in Section 7.1. The formation of PtO_*x*_ or PdO_*x*_ progressively alters the surface state and reduces the coverage of reactive oxygen-containing species (*e.g.*, OH*) as well as glycerol-derived intermediates, thereby hindering continuous adsorption and oxidation processes and leading to catalyst deactivation. Notably, Pd–OH* species are generated only within a limited potential window, whereas higher potentials favour PdO_*x*_ formation, further suppressing the availability of active sites and accelerating activity loss.

### 8.2. Strategies to mitigate catalyst deactivation

To overcome catalyst deactivation arising from surface poisoning and excessive oxidation, both electrochemical and materials-based strategies have been developed to regulate surface states and adsorbate dynamics during glycerol electrooxidation.

From an electrochemical perspective, dynamic potential modulation, such as pulsed potentials, provides an effective means to periodically reset surface oxidation states and suppress the accumulation of strongly adsorbed intermediates. Pulsed electrolysis on Pt catalysts can maintain higher OH* coverage and promote the desorption of poisoning species.^127^ The short duration of GLY oxidation potential (0.7 V *vs.* RHE) prevented the accumulation of poisoning intermediate species and the over-oxidation of the Pt catalyst (Fig. 18a). The application of relatively low potential (0.3 V *vs.* RHE) facilitated the rapid desorption of GLCA, allowing it to diffuse into the solution. The resulting improvement in GLCA selectivity relative to constant-potential operation illustrates how product removal and surface renewal can be coordinated during electrolysis.

**Fig. 18:**
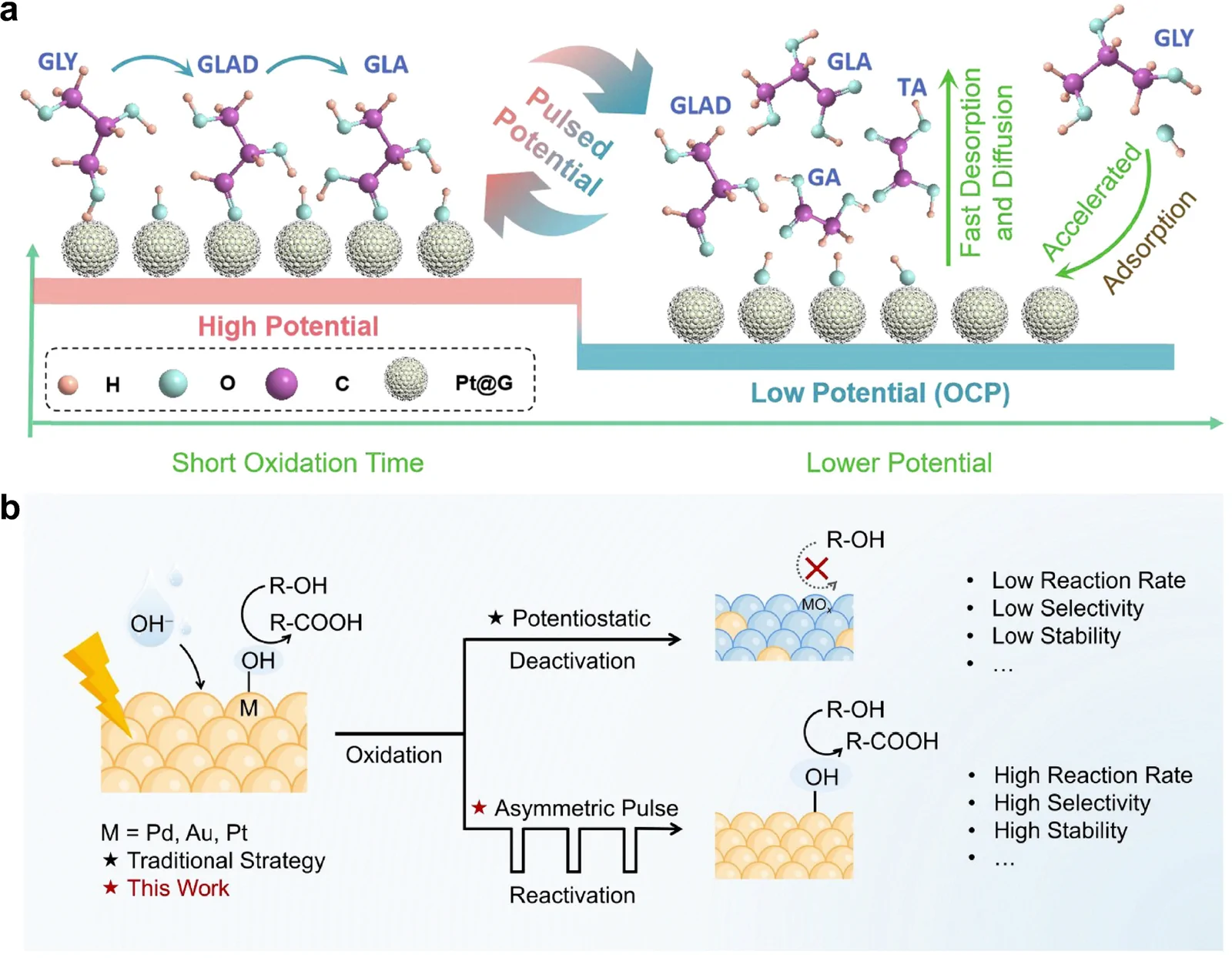
(a) Schematic of the pathways of pulsed electrocatalysis-based GOR. Abbreviations are retained from the original figures: GLA, GA and GLAD correspond to GLCA, GCA and GLD, respectively, in the main text. Reproduced from Chen *et al.*^127^ under the terms of the Creative Commons Attribution 4.0 International License (CC BY 4.0). Copyright 2024 The Authors, published by Springer Nature. (b) Schematic illustration of traditional potentiostatic strategy and asymmetric pulse potential strategy in GOR. Reproduced with permission from Xia *et al.*^134^ Copyright 2024, Wiley-VCH GmbH.

A complementary function is demonstrated by asymmetric pulsing on Pd, where reduction intervals periodically convert PdO_*x*_ back to metallic Pd and restore the surface supporting Pd–OH* formation (Fig. 18b).^134^ Operation beyond 2800 h was reported under the specified pulsing protocol with regular electrolyte renewal. These Pt and Pd examples therefore emphasise different contributions of dynamic operation: limiting adsorbate accumulation and product retention, and reversing an inhibiting surface oxidation state. The pulse parameters and durability conditions are compiled in Table 3.

**Table 3:** Representative catalyst deactivation mechanisms and corresponding mitigation strategies in glycerol electrooxidation

Catalyst	Deactivation mechanism	Mitigation strategy	Test conditions	Representative result	Ref.
Pt@G	Pt surface oxidation and accumulation of strongly adsorbed intermediate species block active sites	Alternate oxidation and low-potential steps to limit Pt oxidation and release adsorbed species	1 M KOH + 20 mM GLY; 0.7 V for 0.05 s/0.3 V for 0.5 s; 10–50 C electrolysis charge	Current retention: 30–60% under pulses *versus* ≤10% at constant potential. GLCA selectivity 81.8–74.4% at 14.7–59.4% glycerol conversion	127
Pd/NF	Pd-OH* is overoxidized to inactive PdO_*x*_, depleting reactive OH* sites	Brief cathodic steps reduce PdO_*x*_ and regenerate the active Pd surface	0.5 M KOH + 0.3 M GLY; 0.8 V for 60 s/0 V for 5 s; electrolyte renewed about every 24 h	>2800 h operation with GLCA selectivity >70%, with an average reaction charge of ∼2000 C per 24 h; constant-potential control nearly inactive within 6 h	134
PtRu/NiFe-LDH^a^	Surface Pt oxidation is the main identified loss mechanism; glycerate adsorption also inhibits activity	Use pulsed electrolysis to reduce PtO_*x*_ and regenerate metallic Pt sites	(a) 1 M KOH + 0.1 M GLY, 0.9 V after pre-oxidation; (b) MEA flow cell, room temperature, pulsed operation	(a) Pt–O fraction: 47.2% after constant potential *versus* 42.7% after pulsing; (b) >120 h operation	170
PtSb_1_/TiO_*x*_	Oxidation of Pt and TiO_*x*_ active sites reduces activity/selectivity	Introduce isolated Sb into Pt to stabilize Pt–Sb bonds and suppress oxidative deactivation	0.5 M K_2_SO_4_ + 0.5 M GLY; pH 7; cell voltage 0.8 V; ten consecutive cycles, 120 h total	GLD selectivity >80% across 120 h	171
PdNiMo-EC	Excessive OH* coverage blocks Pd sites; long-term testing also reveals partial Mo leaching	Combine Ni/Mo tuning of OH* coverage with periodic low-voltage recovery steps	MEA flow cell; 1 M KOH + 0.4 M GLY; room temperature; Cell voltage 1.2 V for 3200 s/0 V for 400 s	About 50 mA cm^−2^ maintained for >1000 h under interval operation; GLCA selectivity >50%; ∼27.3% Mo loss after 1000 h testing	172
PtNi_2_/C	CO and strongly adsorbed glycerol-derived species occupy Pt sites	Pt–Ni alloy electronic regulation optimizes CO*/OH* binding and facilitates oxidation/removal of carbonaceous intermediate	1 M KOH + 1 M GLY; 0.9 V; 2 h chronoamperometry	Final mass activity ∼0.02 A mg_Pt_^−1^, ∼2× PtNi_1_/PtNi_3_; improvement remains limited	91
Pd-CoFe LDH/NF	Strong adsorption of the carbonyl intermediate *CO–CHOH–CH_2_OH inhibits active sites	Couple Pd to CoFe LDH to weaken intermediate binding and supply reactive OH*	MEA flow cell, cell voltage 1.2 V, 1 M KOH + 0.1 M glycerol, 30 °C, 200 h, 25 cm^2^	85% activity retained after 200 h	173
Au-NW@PEI	Strongly adsorbed carbon intermediates poison Au; Au nanowires undergo Ostwald ripening and aggregation	Use a stable polyethyleneimine layer to promote intermediate desorption and limit nanowire coarsening	1 M KOH + 0.5 M GLY; 1.15 V; 5 h chronoamperometry	Current retention after 5 h: 72.8% *versus* 45.1% for Au-NW, normalized to the current at 100 s; separately, 58.8% GLCA selectivity was obtained at 1.15 V after 2 h	174
Au-CeO_2_/CNT	Glycerate product inhibition and Au nanoparticle aggregation reduce activity	Introduce Au–CeO_2_–CNT junctions to weaken glycerate adsorption and restrain Au aggregation and promote OH* formation	1 M KOH + 0.1 M GLY; 1.02 V; 60 °C; 4 h per cycle, 5 consecutive cycles.	Cycles 1 → 5: GLY conversion 62.6 → 56.1%; GLCA yield 30.4 → 22.5%. Au/CNT conversion falls from 65.2 to 2.7%; negligible Au leaching was observed	121
Pt/CeNC	GLCA adsorption inhibits Pt; aggregation of Pt nanoparticles lowers cycling activity	Ce single-atom sites downshift Pt d-band to weaken GLCA binding, promote water activation to OH* and anchor Pt nanoparticles	1 M KOH + 0.6 M GLY; 0.87 V; 60 °C; 15 cycles, 10 h per cycle	GLCA selectivity remains nearly constant across 15 cycles (55–60%); post-test Pt nanoparticles size remains dispersed (1.6 ± 0.4 nm), without marked aggregation	169
MnO_2_/CP	Acidic OER causes lattice-oxygen loss and Mn dissolution, including MnO_4_^−^ formation	Replace OER with glycerol oxidation; adsorbed GLY suppresses destructive oxygen chemistry and Mn overoxidation	0.005 M H_2_SO_4_ + 0.2 M GLY; 10 mA cm^−2^; comparator: the same acid without GLY	>865 h at ∼1.40 V without significant activity loss, *versus* ∼10 h for OER at the same current density. FA FE averaged ∼53% over 96 h	175
CuPd single-site catalyst (CuPd SSC)	Carbonaceous/CO-type intermediate poisoning is proposed for Pd/C; residual activity loss on CuPd SSC is mainly attributed to glycerol/OH^−^ depletion and changes in the electrolyte	Atomically disperse Cu/Pd sites to limit multi-site adsorption and carbonaceous poisoning while promoting hydroxide adsorption; renew the electrolyte to reverse depletion-related activity loss	1 M KOH + 0.5 M GLY; 1.6 V; 144 h chronoamperometry; separate electrolyte-renewal controls	144 h electrolysis with FA FE decreasing from 73% to 60%, ∼24% current density loss over 144 h; activity recovered after electrolyte renewal in separate cycling tests	176

a For PtRu/NiFe-LDH, (a) and (b) denote separate mechanism and durability tests; pre-oxidation in (a) was at 1.35 V for 10 min in 1 M KOH. Complete pulse timings are not specified in the main article.

Beyond electrochemical control, catalyst design and interfacial engineering can mitigate deactivation by regulating intermediate adsorption, facilitating the formation of reactive hydroxyl species and stabilizing active sites. In the Pt/CeNC catalyst system, Ce single-atom sites downshift the Pt d-band centre, weakening GLCA adsorption and alleviating product inhibition.^169^ These sites also promote water activation to generate OH* and anchor Pt nanoparticles, thereby limiting aggregation during repeated electrolysis. Consistent with these effects, GLCA selectivity remained approximately 55–60% over 15 consecutive cycles of 10 h each, while post-test characterization showed that the Pt nanoparticles remained well dispersed.

Table 3 summarises reported deactivation mechanisms, mitigation strategies and durability results, extending the discussion to particle aggregation and metal leaching. These comparisons highlight the need to distinguish catalyst degradation from reversible activity loss associated with changes in electrolyte concentration. Durability should therefore be evaluated alongside product selectivity, regeneration procedures and electrolyte-renewal protocols.

Together, sustained GOR requires the removal of inhibiting adsorbates while maintaining or restoring the active surface state. Because different loss mechanisms can produce similar current decay, mitigation strategies should be matched to evidence identifying the cause. Product analysis, recovery tests and catalyst characterisation are therefore needed to assess whether a strategy sustains the desired pathway as well as electrochemical activity.

## 9. Conclusion and perspectives

### 9.1. Stepwise organization of the GOR reaction network

Glycerol electrooxidation is governed by a coupled network of electrochemical surface steps and chemical reactions in the electrolyte. Adsorption and initial dehydrogenation establish the early branching towards GLD and DHA, but this branching does not alone determine the final product distribution. After their formation, C3 intermediates may remain adsorbed and undergo further oxidation, desorb and react in the electrolyte, or return to the catalyst surface through readsorption. The product distribution observed after electrolysis therefore reflects the relative contributions of these routes. This stepwise framework links changes in catalyst structure and reaction conditions to the reaction steps they affect and the products that follow. Within this framework, DFT evaluates the energetic plausibility of elementary steps, *in situ*/*operando* spectroscopy identifies intermediates and catalyst states during electrolysis, and isotopic labelling traces carbon through competing pathways. Together, these approaches allow proposed pathways to be evaluated against reaction energetics, intermediates observed during electrolysis and isotopically traced carbon flow.

### 9.2. Systematic principles governing GOR selectivity and stability

Systematic comparison across the GOR network identifies three linked balances that govern selectivity and stability: C- *versus* O-bound adsorption, formation of reactive surface species *versus* passivation, and intermediate retention *versus* removal. C-bound adsorption involving adjacent metal sites increases the probability of C–C cleavage and strongly adsorbed fragments. O-bound or single-carbon adsorption is more compatible with C3 retention. OH* promotes dehydrogenation, but further surface oxidation at higher potentials can drive deeper oxidation and reduce the availability of active sites. Intermediate retention allows consecutive surface oxidation, but prolonged retention can also promote C–C cleavage and site blocking. Desorption limits these surface processes while exposing intermediates to chemical conversion in the electrolyte.

The catalytic environment couples these balances through its influence on adsorption, OH* formation and intermediate behaviour. Their regulation should therefore serve a specified reaction sequence, rather than maximise adsorption strength or oxidative activity independently. This provides a basis for connecting catalyst composition, interfacial functionality and operating conditions with the steps required to form and preserve the target product.

### 9.3. Mechanistic gaps and perspectives for catalyst design and operation

Although the main GOR routes have been identified, important evidence gaps remain in characterising surface processes, assigning pathway branches and distinguishing catalyst deactivation mechanisms. For surface processes, assignments of C- or O-bound adsorption, OH* formation and site-specific C–C scission often rely on idealised DFT surfaces. Their validity for adsorbate-covered and potential-dependent catalyst structures has not been established across catalyst systems. Spectral bands compiled from different catalysts and electrolytes should likewise be treated as reference ranges rather than universal fingerprints. For pathway assignment, GLD/DHA interconversion means that their measured distribution does not directly report the initial dehydrogenation branch. The relative contributions of electrochemical C–C scission on the surface and chemical cleavage after desorption, including possible retro-aldol pathways, also remain unresolved. For catalyst deactivation, activity loss can arise from adsorbate accumulation, surface oxide formation or structural change, but their separate contributions cannot be inferred from current decay alone. The potential-dependent transition from reactive OH* coverage to more oxidized surface states has not been described by a transferable relationship across catalyst classes.

Designing the catalytic environment should connect each modification to the reaction step it is intended to regulate. Where multiple carbon-metal bonding favour unwanted fragmentation, alloying or surface modification can restrict these configurations while retaining sites for glycerol activation. Hydroxyl-rich interfaces can enhance local glycerol availability, but excessive adsorbate coverage should be avoided to preserve sites for OH* formation and subsequent dehydrogenation. Interfacial binding should then permit the required intermediate conversion without causing persistent retention or product inhibition. Once the target product forms, its release should be favoured to limit further oxidation and renew surface sites. For C1 targets, the same framework requires promoting the necessary cleavage steps while limiting further product oxidation.

Operating conditions should maintain the surface chemistry required for the target pathway. Potential should be selected within a window established by product analysis that supports the necessary active surface state while limiting competing oxidation and passivation. Electrolyte pH and composition should enable the required activation and chemical steps while limiting unwanted product degradation. Reactant supply and product removal should be adjusted to prevent depletion and excessive product exposure. Regeneration strategies should address the identified cause of inhibition while preserving or restoring the catalytically active surface state.

Future studies should evaluate the proposed design strategies and operating conditions through complementary kinetic, *operando* and reaction-probe measurements during sustained operation. At practical current densities, concentration gradients, gas evolution and catalyst restructuring may alter the pathways observed at lower current densities. Validation should therefore establish whether the proposed active states and target product selectivity can be maintained under these conditions. Practical implementation also requires integrating GOR anodes with HER, CO_2_RR or other cathodic reductions. Such integration requires compatible operating conditions for both half-reactions, adequate mass transport, efficient product separation, and control of electrolyte composition and membrane crossover. These device-level considerations are discussed in dedicated reviews and perspectives.^12,51,177^ Establishing how full-cell operation affects GOR pathways will help translate mechanistic understanding into catalyst designs that sustain target-product selectivity, activity and stability.

## Conflicts of interest

There are no conflicts to declare.

## Data Availability

No primary research results, software or code have been included and no new data were generated or analysed as part of this review.
